# Genome-wide association analysis of nutrient traits in the oyster *Crassostrea gigas*: genetic effect and interaction network

**DOI:** 10.1186/s12864-019-5971-z

**Published:** 2019-07-31

**Authors:** Jie Meng, Kai Song, Chunyan Li, Sheng Liu, Ruihui Shi, Busu Li, Ting Wang, Ao Li, Huayong Que, Li Li, Guofan Zhang

**Affiliations:** 1 Key Laboratory of Experimental Marine Biology, Institute of Oceanology, Chinese Academy of Sciences, Qingdao, 266071 Shandong China; 2 Laboratory for Marine Biology and Biotechnology, Qingdao National Laboratory for Marine Science and Technology, Qingdao, 266071 Shandong China; 3 Laboratory for Marine Fisheries and Aquaculture, Qingdao National Laboratory for Marine Science and Technology, Qingdao, 266071 Shandong China; 4 National & Local Joint Engineering Laboratory of Ecological Mariculture, Qingdao, 266071 Shandong China; 5 Center for Ocean Mega-Science, Chinese Academy of Sciences, Qingdao, 266071 China; 6 University of Chinese Academy of Sciences, Beijing, 100039 China

**Keywords:** Oyster, Nutrient traits, Genome resequencing, Population structure, Genome-wide association study, Genetic network

## Abstract

**Background:**

Oyster is rich in glycogen and free amino acids and is called “the milk of sea”. To understand the main genetic effects of these traits and the genetic networks underlying their correlation, we have conducted the whole genome resequencing with 427 oysters collected from the world-wide scale.

**Results:**

After association analysis, 168 clustered significant single nucleotide polymorphism (SNP) loci were identified for glycogen content and 17 SNPs were verified with 288 oyster individuals in another wide populations. These were the most important candidate loci for oyster breeding. Among 24 genes in the 100-kb regions of the leading SNP loci, cytochrome P450 17A1 (CYP17A1) contained a non-synonymous SNP and displayed higher expressions in high glycogen content individuals. This might enhance the gluconeogenesis process by the transcriptionally regulating the expression of phosphoenolpyruvate carboxykinase (PEPCK) and glucose 6-phosphatase (G6Pase). Also, for amino acids content, 417 clustered significant SNPs were identified. After genetic network analysis, three node SNP regions were identified to be associated with glycogen, protein, and Asp content, which might explain their significant correlation.

**Conclusion:**

Overall, this study provides insights into the genetic correlation among complex traits, which will facilitate future oyster functional studies and breeding through molecular design.

**Electronic supplementary material:**

The online version of this article (10.1186/s12864-019-5971-z) contains supplementary material, which is available to authorized users.

## Background

The Pacific oyster *Crassostrea gigas* is an important marine fishery resource cultivated globally. Its annual global production continues to increase at a rate of 4 million tons per year [1]. However, with the increasing demand for the high quality of seafood, breeders have began to focuse on the genetic breeding to improve oyster quality traits. Oyster is rich in taurine (50 μmol/g wet weight), amino acids (45–57% of dry weight), and glycogen (20–40% of the dry weight), but with low content of fat and cholesterol [2]. These traits are related not only to the flavor and the quality of oysters, but also to their hardiness, which is the most important objective of nutrient breeding of oysters. More important, many studies revealed that different oysters individuals carrying a large amount of phenotype (glycogen, amino acids contents) and genetic variations, providing important resources to improve oyster qualities [3, 4]. However, the traditional cross breeding methods usually have long cycle and was low efficiency [5]. Recently, molecular breeding has been used for the cultivation of new shellfish varieties. However, this breeding method needs to understand the genetic architecture underlyging these breeding traits [6, 7]. Therefore, the genetic studies of oyster nutrient traits, such as glycogen, protein and amino acids, is necessary for the development of molecular breeding inoysters.

Studies on the genetic basis of glycogen, amino acid, and protein content have been extensively conducted in various domesticated animals, as well as in crops [8–10], cattle [11], pigs [12], Atlantic salmon [13], carp [14], and Asian seabass [15]. However, in bivalves, nutrient trait-related studies are limited. To date, genetic studies on target traits and breeding have mainly focused on their growth and stress resistance [16–18]. Genetic studies of glycogen, protein, and amino acids in mollusks are scarce as they are expensive and not amenable to high-throughput assays. Several SNP loci for glycogen content were detected by target sequencing [19, 20]. For example, an effective haplotype of glycogen synthase is found in *C. gigas*, which is significantly related to the glycogen content [20]. However, these analyses mainly focused on several genes involved in glycogen synthesis and degradation pathways. With respect to complex regulation mechanisms, this analysis cannot detect the major loci and genes.

Recently, more and more evidences revealed that genome-wide association (GWA) studies was a powerful method to identify the nucleotide polymorphisms associated with agronomics traits in plants and animals [21, 22]. In bivalves species, GWAS for growth [23], shell color [24], sex-determination [25] and disease resistance has been conducted in oysters, scallops, etc. Several methods that utilize NGS for genotyping, such as reduced genome representation sequencing methods—2b-RAD-seq, genotyping-by-sequencing (GBS), and whole-genome resequencing analysis [26]. For example, GBS sequencing has been used to conduct GWAS analysis to illustrate the genetic basis of carotene content in scallops [24]. However, reduced genome representation sequencing can genotype only a fraction of the genome. With the advances in high-throughput sequencing technologies, the genome of several mollusks have been completed [27–29]. Therefore, the GWAS of glycogen, protein, and amino acids by genome resequencing will provide valuable information for molecular breeding of oysters, which has not been conducted in marine bivalves until now.

Several traits exhibit heritable covariation, adding to the complexity of breeding. However, information on specific trait is insufficient for molecular breeding. In plants, the correlation analysis of complex traits has been conducted by many studies [30, 31]. In oysters, the objective of breeding is to improve yield, and multiple selection criteria, including glycogen, protein, and amino acids content, have been applied. These traits exhibit positive or negative correlation with each other. Information on trait covariation is essential for the genetic improvement of multiple complex traits [32].

In the present study, we aimed to understand the main genetic effects of nutrient traits and genetic networks underlying phenotypic correlation in oysters. Four hundred and twenty-seven individuals were collected to conduct genome resequencing, which were used to perform GWAS to identified new SNP loci for these characteristics. The candidate SNP loci were further verified with the 190 K SNP array using other populations and the candidate genes were used for the functional analysis. Further, genetic networks between different traits were also analyzed. The results will facilitate future breeding of oysters to improve these traits through genomic selection.

## Results

### Genotyping of 427 individuals from 26 sample collection sites

**Fig. 1:**
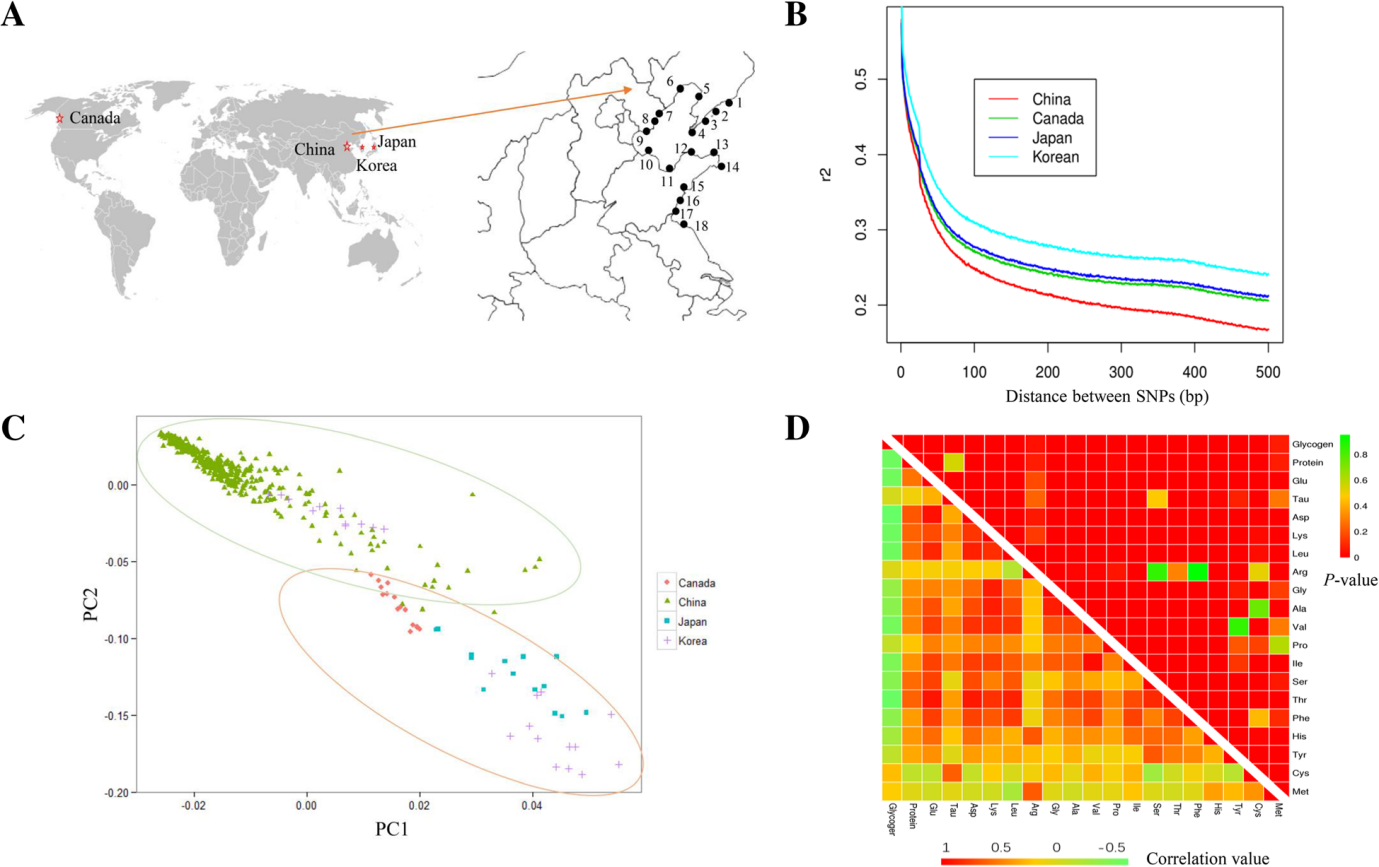
Geographic distribution and genetic structure of 427 oyster individuals. **a**. The world-scale geographic distribution of *Crassostrea gigas*. 1. DanDong; 2. ZhuangHe; 3. Haiyang Dao; 4. DaLian; 5. Bayu Quan; 6. JinZhou; 7. QinHuangdao; 8. ChangLi; 9. LaoTing; 10. BinZhou; 11. WeiFang; 12. YanTai; 13. WeiHai; 14. RongCheng; 15. Qingdao_ShenTanggou; 16. QingDao_JiaoNan; 17. RiZhao; 18. LianYungang. The maps were download from http://commons.wikimedia.org/wiki/Main_Page. **b**, **c**. The LD decay and PCA analyses of the 427 oysters collected. **d**. The correlation analysis of different quality traits in oysters—glycogen, protein, and amino acids content. The x-lab and y-lab indicate correlation value and *P*-value, respectively

In the present study, we collected 427 individuals from different geographical populations (Fig. 1a), covering the main oyster production areas in North China, as well as from Korea, Japan, and Canada. The major information for these 26 locations were represented in Additional file 1: Table S1. Resequencing and population structure analysis of 371 wild oysters collected in China have been conducted by us previously [33]. In the present study, 42 other individuals collected from different countries, including Canada, Japan, and Korea, were further used for whole genome resequencing. In total, 4.8 billion paired-end reads of 427 individuals were generated with a mean depth of approximately 20× for each oyster (Additional file 2: Table S2). After mapping against the reference genome and SNP calling, a total of 52,142,764 SNPs and 12,153,683 small indels (≤ 6 bp) were identified (Additional file 2: Table S2). The accuracy of the identified SNPs was more than 96.5 and 98.2% according to SNP chip array and Sanger sequencing verification [33].

These SNPs were used to evaluate linkage disequilibrium (LD), which decayed to 0.25–0.33 beyond 100 bp in different populations (Fig. 1b). This LD decay was faster than that in several plants (such as rice [34], maize [35], soybean [31], and cotton [36]) and mammals (such as pig [37] and human [38]). For the fast decay of LD, it is easy to locate the candidate genes within a LD block, a universal problem that occurs in plant GWAS [31, 39]. The population of *C. gigas* in North China can be classified into four main clades, based on their geographical distribution [33]. In the present study, the neighbor-joining tree (NJ) suggested that three populations from Canada, Japan, and Korea were different from the individuals in China (Additional file 10: Figure S1). The *C. angulata* was used as the outgroup. The PCA analysis further supported this difference (Fig. 1c). This was related to their geographical distribution pattern, which has been reported to be associated with some special polymorphism loci in mollusks. This suggests that the 427 individuals may have population differentiation, which was used as a covariate within the GWAS model.

### Phenotyping of 427 individuals

The 427 female individuals collected from different locations were usd for breeding and produce 427 half-sib families. The F_1_ oysters from 427 half-sib families were bred and cultured in Qingdao and were used for the assay of their phenotype traits (the content of glycogen, protein, and 18 amino acids). The 30 individuals were collected in each family and were used for phenotype detection (a total of 12,810 individuals), which represented 427 female parents’ phenotypic values. The F_1_ breeding method and phenotypic values have been presented in Additional file 11: Figure S2 and Additional file 3: Table S3, respectively. Except the content of methionine (Met), arginine (Arg), and cysteine (Cys), other traits presented normal distribution (Additional file 12: Figure S3) and exhibited significant variations among the 427 individuals (Additional file 4: Table S4). The glycogen content ranged from 44.02 to 239.04 mg/g and the largest fold change was 5.43. For most amino acids, the fold changes among individuals ranged from 1.4 to 3.9. However, the two S-containing amino acids, including Cys and Met, presented 19.4- and 18.4-fold changes. The phenotypic diversity of these traits was comparable with that of various crop varieties reported previously [40]. Among the eighteen detected amino acids, the content of glutamic acid (Glu) and taurine (Tau) was the highest, accounting for 14 and 11% of the total amino acid content (TAA), respectively. Besides, the delicious amino acids (DAAs), including Glu, aspartate (Asp), glycine (Gly), alanine (Ala), phenylalanine (Phe), and tyrosine (Tyr), accounted for 41% of the TAA content (Additional file 13: Figure S4). We also estimated phenotypic correlations among the analyzed traits. The glycogen content exhibited significant negative correlation with traits such as protein and amino acid component (except, Cys and Met). Further, 15 amino acids showed significantly positive correlation with each other (Fig. 1d, Additional file 5: Table S5). Especially, Asp, Leu, Glu, and Thr presented strong positive correlation (*r* > 0.8, *P* < 0.01) with each other, as they are physiologically correlated. However, the content of Met exhibited negative correlation with several amino acids, but it positively correlated with the content of Cys, Arg, His, and Try.

### GWAS identified the significantly associated loci (SAL) with glycogen and protein

**Fig. 2:**
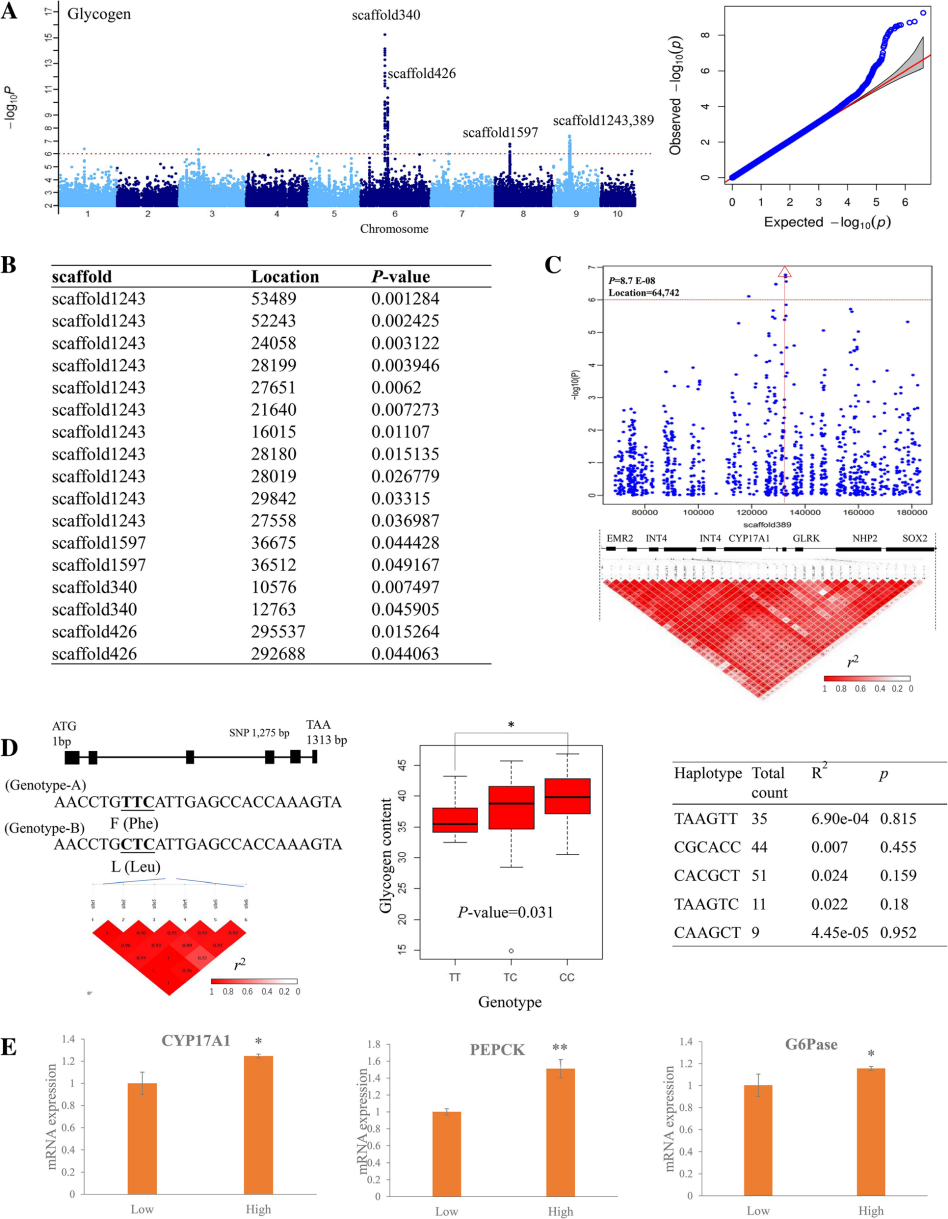
Genome-wide association study of glycogen content and candidate gene analysis in different oyster populations. **a** The Manhattan and QQ plots of glycogen content in oysters. The negative log_10_-transformed *P*-values from a genome-wide scan are plotted against position on each of the 10 chromosomes. Red horizontal dashed line indicates the genome-wide significance threshold. **b** The verification of significant loci using 190 K SNP chips with 300 individuals collected from Qingdao in 2013. **c** The 0.1-Mb region on each side of the peak SNP in scaffold389, and the position of peak SNP is indicated by a vertical red line with the red triangle. **d** The gene base analysis (GBA) of CYP17A1 by the PCR with 100 individuals collected from Qingdao in 2013. After the identification of SNPs, the association analysis was conducted using the SHEsis software (http://analysis.bio-x.cn/) and five haplotypes were identified. Furthermore, the association between the SNP loci or related haplotypes and glycogen content was analyzed. **e** The expression pattern of CYP17A1, PEPCK, and G6Pase in high and low glycogen content populations. Significant difference was calculated by the *t*-test (** indicates *P* < 0.01, * indicates *P* < 0.05, *n* = 15)

We conducted a GWAS on 20 traits (including the content of glycogen, protein, and 18 amino acids) based on 4.2 million high quality SNP markers (SNPs with a minor allele frequency [MAF] of ≥0.05) genotyped from 427 individuals using the mixed linear model (Additional file 14: Figure S5, Additional file 15: Figure S6, Additional file 16: Figure S7). The population structure was represented by the first three principal components, which were fitted as fixed effects. After GWAS, we identified 175 and 32 significantly associated loci (SAL) for glycogen and protein content, explaining 3.59–23.72% and 3.10–16.61% of phenotypic variation with a suggestive threshold (*P* < 1 × 10 ^− 6^ in a mixed model; false discovery rate (FDR) < 0.05). For glycogen analysis, 168 of 175 SNPs were clustered into a peak on chromosomes 6, 8, and 9 (Additional file 6: Table S6, Fig. 2a), which were mainly discussed in the following parts. Sixty-seven SNPs were clustered in scaffold1243 (chromosome 9), 7 loci in scaffold1597 (chromosome 8), 39 loci in scaffold340 (chromosome 6), 16 loci in scaffold389 (chromosome 9), and 39 loci in scaffold426 (chromosome 6) (Additional file 6: Table S6). Among these loci, 111 SNPs distributed in the gene region and 57 in the intergenic region. Also, these clustered loci within the same scaffold presented high LD correlation (*r*^2^ > 0.4) (Figs. 2b, Additional file 15: Figsure S6 and Additional file 16: Figure S7). For protein contents, 21 of 32 SNPs were clustered into a peak on scaffold340 (chromosome 6).

### GWAS signals validation for glycogen and protein contents with 288 individuals

We further conducted the association analysis with 288 oyster individuals collected in Qingdao in 2014 to validate the GWAS results for glycogen and proteins. These 288 individuals were one-year old and were cultured in the same environment, which were used for phenotype and genotype detection. The genotypes were obtained using the 190 K SNP Chips [41]. After SNP filtering, 145,550 SNPs were used for GWAS analysis. A total of 9,050 and 9,336 SNP loci were identified to be association with glycogen and protein contents (*P-value* ≤ 0.05) (Additional file 7: Table S7, Additional file 8: Table S8). We mainly focused on the SNPs located on candidate regions (scaffold1243, 1597, 340, 389 and 426) which obtained from the GWAS results. We found 17 loci on scaffold1243, scaffold1597, scaffold340, and scaffold426 were further verified to be significantly (*P-value* ≤ 0.05) associated with glycogen content (Fig. 2b). Four loci in scaffold340 were verified to be associated with protein contents (*P*-value ≤ 0.05). For the high LD values (*r*^*2*^>0.4) of SNPs on scaffold1243, 1597, 340 and 426 (Additional file 15: Figure S6), we think that these regions were most important candidates for glycogen analysis.

### CYP17A1 was responsible for glycogen synthesis

We have conducted the candidate genes analysis within 100 kb of the leading SNPs on scaffold1243, 1597, 340, 389 and 426. There are 21 genes located within 100 kb of the leading SNPs in these regions based on our genome sequence (Additional file 17: Figure S8). Only two genes, including steroid 17-α-hydroxylase/17,20 lyase-like (CYP17A1) and one unknown gene, contained non-synonymous SNPs. As the major candidate gene associated with glycogen contents, we have analyzed CYP17A1 at a single gene resolution level in oysters.

CYP17A1 contained 12 significantly associated SNPs with glycogen content and 8 SNPs located in exon regions. A significant non-synonymous SNP that caused a change from C (11 individuals) to T (341 individuals) at 425 amino acid position in the protein sequence (1,275 bp in the CDS) resulted in a change from F (Phe) to L (Leu), accounting for 6.2% of the phenotypic variation (Fig. 2c, d). Therefore, there was an average increase of 135.77 mg/kg in glycogen content, which is higher than the average effect of unfavorable allelic class (108.65 mg/kg). According to the multiple alignment and three-dimensional protein structural modeling analysis, F425 L located at a turn in β-sheet and formed the substrate-binding pocket (Additional file 18: Figure S9). This mutation might affect the substrate binding activity of this enzyme. Further, we have selected 100 individuals collected in Qingdao in 2014 to conduct the GBA analysis. This non-synonymous SNPs were also associated with glycogen content within this population (*P-value* = 0.03) (Fig. 2d). Also, we have identified a total of 19 SNPs in the CDS region of this gene and four haplotypes based on this non-synonymous SNPs (Fig. 2d). However, these haplotypes did not exhibit significant association with glycogen content. Gene expression analysis showed that the CYP17A1 was highly expressed in individuals with higher glycogen contents (Fig. 2e). In order to observe its regulation mechanism for gluconeogenesis metabolism, we have analyzed two key genes expressions participated in gluconeogenesis metabolism process, including PEPCK and G6Pase. Their expressions were higher in high glycogen contents individuals, indicating their stronger gluconeogenesis capacity.

### GWAS analysis of different amino acids content

**Fig. 3:**
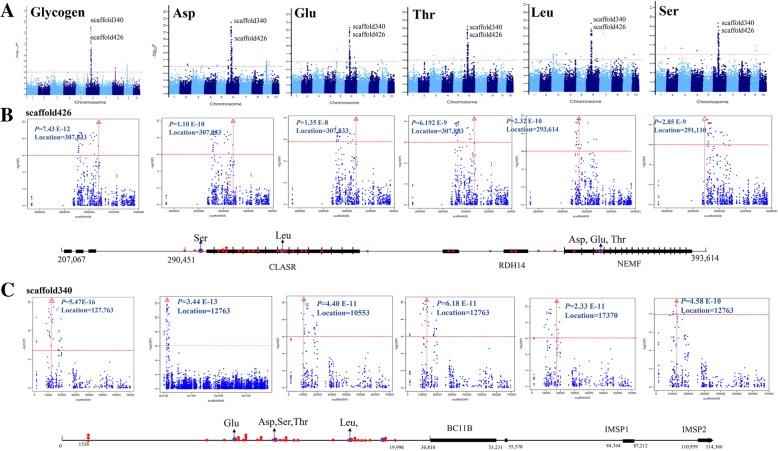
Genome-wide association study of amino acids, including Asp, Glu, Leu, Lys, Ser, Thr, and Val. **a** The Manhattan and QQ plots of these traits. The negative log_10_-transformed *P-*values from a genome-wide scan are plotted against position on each of the 10 chromosomes. Red horizontal dashed line indicates the genome-wide significance threshold. **b, c** The upper panel indicates the 0.1-Mb region on each side of the peak SNP in scaffold426 and scaffold340, and the position of peak SNP is indicated by a vertical red line with the red triangle. The bottom panel shows the annotated genes of the 100-kb region. Significant SNP that surpassed the threshold is indicated by red plots and peak SNP is indicated by purple plots. The dotted lines with arrows represent the traits related to the peak SNP. Gene is indicated by black boxes

We have also conducted GWAS analysis with 426 individuals with 18 different amino acids. A total of 787 significant SNPs (*P* < 10^− 6^) were identified to be associated with these traits (Additional file 6: Table S6), explaining 0.3–49% of phenotypic variation. The largest was observed for Arg content in oysters. The Manhattan plots revealed that 417 clustered SNPs were distributed in different chromosomes (Additional file 14: Figure S5, Additional file 6: Table S6). For the high correlation among amino acid components, many significant SNPs for different amino acids were overlapped (Additional file 6: Table S6). Especially, we identified two regions in scaffold426 and scaffold340 co-associated with the content of Asp, Glu, Leu, Ser, and Thr (Fig. 3a), and their phenotype correlation coefficient was > 0.8. Furthermore, these two regions overlapped with glycogen content, indicating that the same underlying mechanisms regulated the content of glycogen and these five amino acids. Among these two regions, 10 genes were identified, and they were the most important candidates to explain their genetic correlation (Fig. 3b and c). Also, a total of 66 candidate genes for all amino acids’ components were identified within 100 Kb regions (Figs. 2 and 3, Additional file 19: Figure S10).

**Fig. 4:**
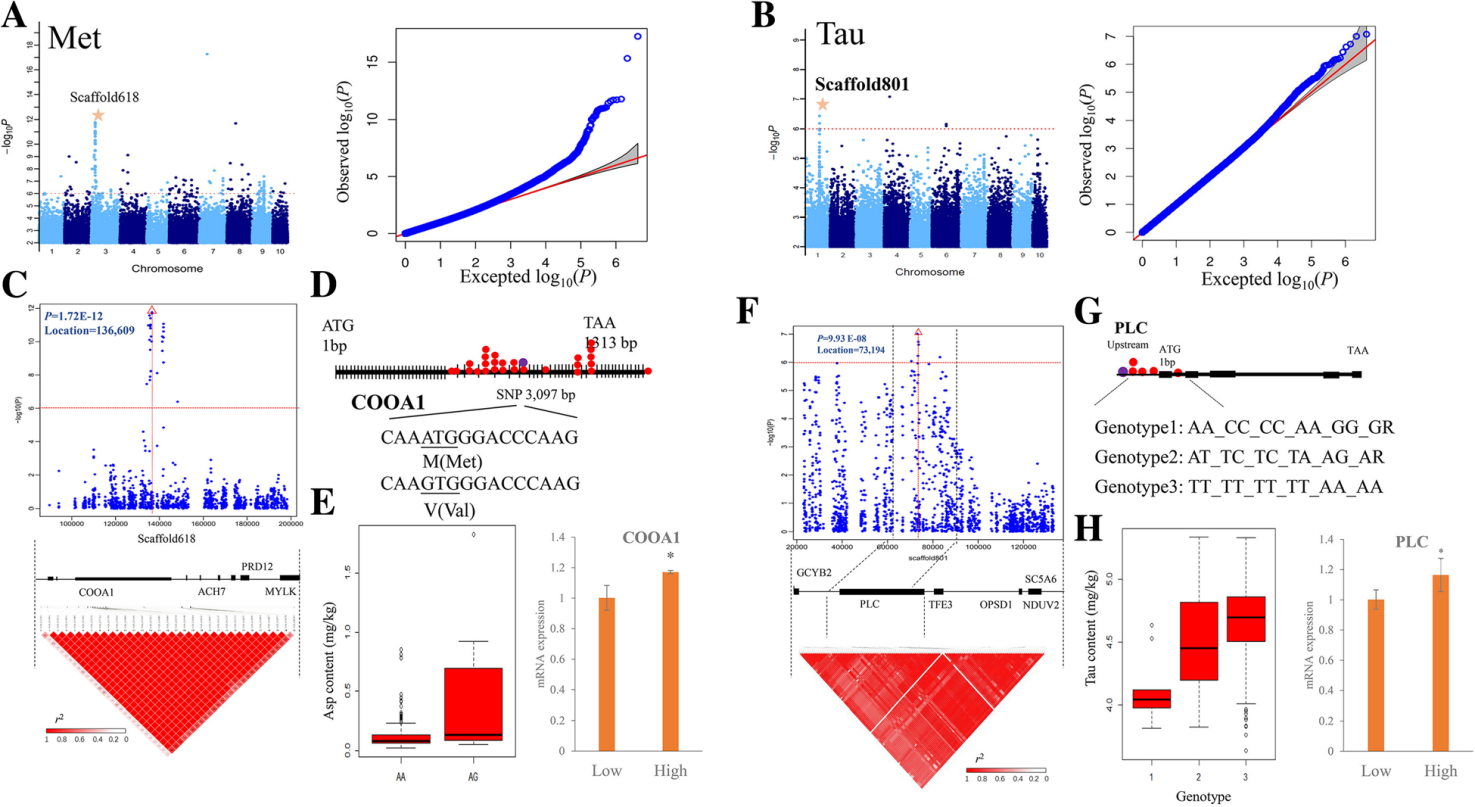
Genome-wide association study of Met and Tau with 427 individuals. **a**, **b** The Manhattan and QQ plots of Met and Tau content. The negative log10-transformed *P*-values from the genome-wide scan are plotted against position in each of the 10 chromosomes. Red horizontal dashed line indicates the genome-wide significance threshold. **c**, **f** The 0.1-Mb region on each side of the peak SNP in scaffold618 (**c**) and scaffold801 (**f**), the position of peak SNP is indicated by a vertical red line with red triangle. The bottom panel shows the annotated genes of the 100-kb region and the LD analysis of pairwise SNP loci in the 100 kb-region on both sides of the leading SNP obtained by GWAS (*P* < 2 × 10^− 4^). **d** The candidate genes for Met content with one non-synonymous mutation in the coding region. **e** The left panel indicates the Met content in different genotypes of *COOA* gene. The right panel indicates the mRNA expression of *COOA* genes in high and low Met content oysters. Different letters indicate the significant differences (*P* < 0.05) and the error bars represent ± SD (*n* = 15). **g** The candidate genes for Tau content and different haplotypes with five significant SNP loci. **h** The left panel indicates the Tau content in different haplotypes of *PLC* genes. The right panel indicates the mRNA expressions of *PLC* genes in high and low Tau content oysters. Different letters indicate significant differences (*P* < 0.05) and error bars represent ± SDs (*n* = 15)

Besides, Met exhibited a weak negative correlation with others (except with Cys), which was further used to analyze (Additional file 5: Table S5). Out of 154 signals in GWAS for Met content, 60 were clustered in chromosomes 3, 6, and 9. These clustered SNPs were distributed in the following regions: 134,231 to 148,283 bp in scaffold618 (chromosome 3), 50,589 to 63,179 bp in scaffold535 (chromosome 9), 47,819 to 51,456 bp in scaffold1610 (chromosome 7), and 242,446 to 243,557 bp in scaffold142 (chromosome 3) (Fig. 4a, Additional file 6: Table S6). In these regions, only two genes contained non-synonymous SNPs. One was a non-synonymous SNP mutation from G to C (GWAS, *P* = 7.98E-07) at 548-bp position of an unknown gene (*Crassostrea*_*gigas*_GLEAN_10005147) induced amino acid transformation from T to R. The other was a non-synonymous SNP mutation from A to G (GWAS, *P* = 2.03E-08) at 3097-bp position of collagen α-1(XXIV) chain gene (*Crassostrea*_*gigas*_GLEAN_10013041) induced amino acid transformation from M to V (Fig. 4b). Moreover, these two SNPs induced 6.55 and 15.75% phenotypic variations, respectively, which were the most important candidate genes involved in Met synthesis (Additional file 6: Table S6). Another S-containing amino acid, Tau is a FAA, which is not utilized in protein synthesis, but is found free or in simple peptides. Taurine is synthesized from Met and Cys, and it is present in high concentrations in oysters and is the most abundant FAA. In the present study, 12 significant SNP signals were identified for taurine content (Fig. 4c). Among them, six signals were clustered on scaffold801 (Chromosome 1), located upstream and in the extron of perlucin (PLC) (Fig. 4d), explaining the phenotypic variation by 7.54–8.48% (Additional file 6: Table S6). Besides, a significant correlation between the polymorphisms of these two genes (PLC and collagen α-1) and two S-containing amino acids content (Tau and Met) was observed, inducing a differential expression pattern of these two genes in high and low amino acid content individuals (Fig. 4c, d and h). It has been revealed that PLC and collagen α-1 could promote calcium carbonate precipitation and participate in shell formation in mollusks. Also, these two S-containing amino acids participated in shell formation and biomineralization process in mollusks [42, 43]. In the present study, the polymorphism of these two genes induced their differential expression patterns, which might affect the shell formation process in oyster. However, further studies should be conducted to analyze this genes function in the amino acids’ metabolism process.

### Complex trait interaction

**Fig. 5:**
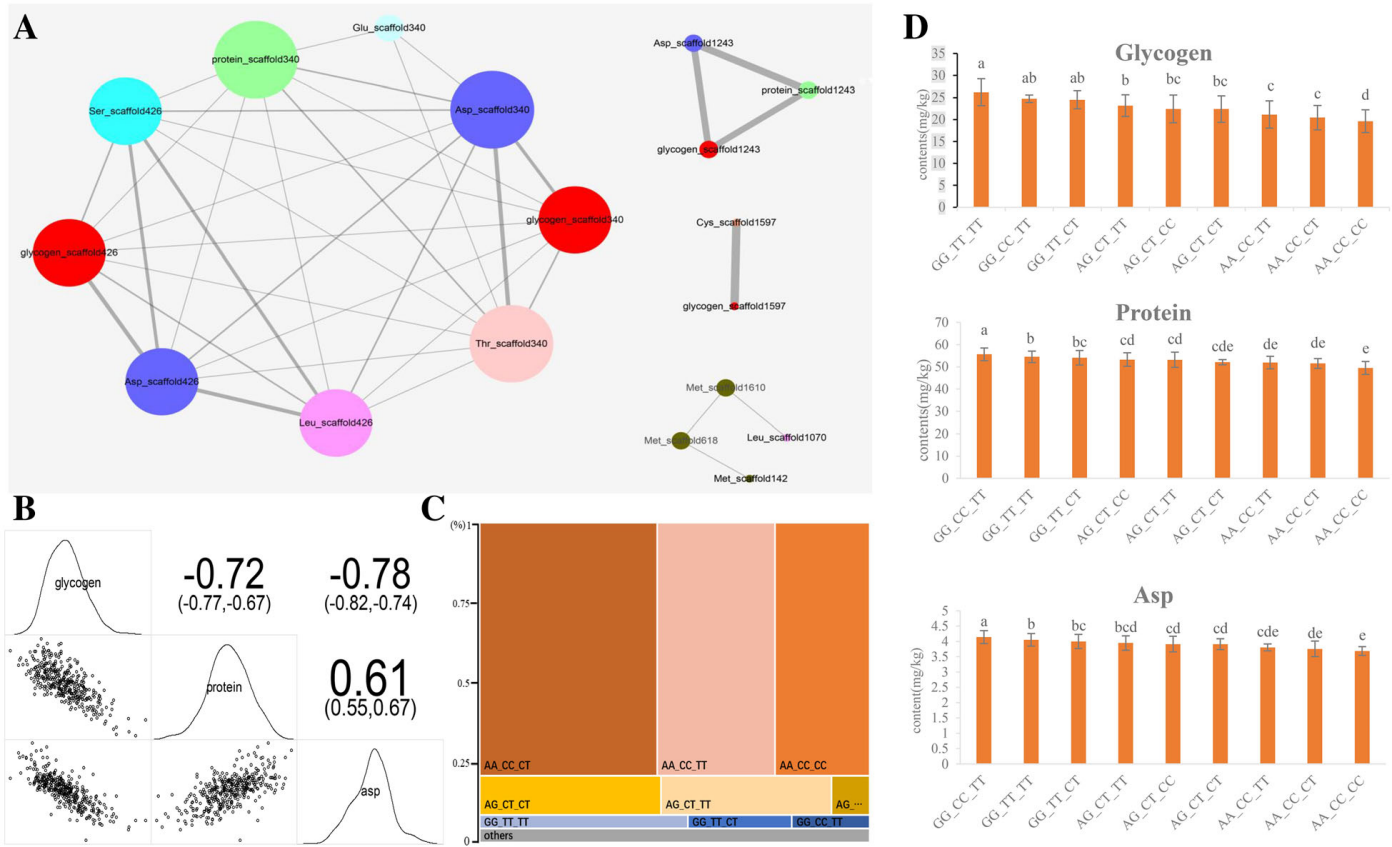
Association networks among different traits in oysters**. a** The networks were constructed with different traits using Cytoscape. The node represents traits and their responsible SAL. The edges between the SALs from different traits are linked by LD. The edges with an average LD ≥ 0.1 are displayed. **b** The correlation analysis among glycogen, protein, and Asp content in 427 different oysters. The values indicate the correlation among different traits. **c** The number of oysters distributed among different genotypes of the peak SNPs in scaffold340, scaffold426, and scaffold1243. **d** The content of glycogen, protein, and Asp in different genotypes. Different letters within the same trait indicate significant differences (*p* < 0.05) and the error bars represent SDs

From the physiological correlation analysis, we found that several traits displayed positive or negative correlation with each other, suggesting that they might be genetically co-regulated (Additional file 5: Table S5). To elucidate the genetic basis of the correlation among different traits, we analyzed the association networks using by a previously reported method [31] based on the LD analysis. The results revealed that several SALs were connected for most traits, which regulated the association phenotypes (Additional file 9: Table S9, Fig. 5a). One group of the connected SALs located on scaffold340 and scaffold426 regions, which regulated the content of glycogen, protein, Asp, Ser, Leu, and Thr. Besides, the SAL on scaffold1243 plays important roles in the regulation of glycogen, protein, and Asp content, whereas, that on scaffold1597 regulates glycogen and Cys content.

Especially, in oysters, the content of glycogen, protein, and Asp has significant positive or negative correlation with each other (Fig. 5b). We have identified three node SNP loci associated with these three characters in scaffold340, scaffold426, and scaffold1243. To verify the effects of these three regions in the association network, we analyzed different genotypes with leading SNP loci in scaffold340, scaffold426, and scaffold1243 (Fig. 5c). The content of glycogen, protein, and Asp was compared between different genotyping types. The results showed that the oysters with the genotype GG_TT_R (82% individuals) have significantly higher glycogen content and lower protein and Asp content than those with the genotype AA_CC_R (13% individuals), confirming that these three loci play an important role in regulating these three important agronomic traits simultaneously (Fig. 5d). In previous studies, several polymorphic sites have been identified to be associated with glycogen content in mollusks. However, the correlation between different quality traits and their genetic basis has not been studied. Our results provide a genetic explanation for the significant (*P* < 0.01, Pearson’s product-moment correlation) positive relationship between protein and Asp content, as well as the negative relationship between glycogen and protein or Asp content.

## Discussion

We have performed an integrated GWAS analysis with expression profiling data and GBA, which we used to rapidly identify candidate genes associated with glycogen and amino acids contents in oysters. We have mapped 175, 32 and 787 GWAS signals significantly associated with glycogen, protein and types amino acids contents. A total of 17 GWAS signals in four genomics regions (scaffold1243, 1597, 340 and 426) were further validated to be with glycogen contents in another wild population cultured in Qingdao (288 individuals). For the high LD of SNPs in these four regions, we think these were most important candidate regions for glycogen content. Further, gene function analysis found that cytochrome P450 17A1 (CYP17A1) contained a non-synonymous SNP between high and low glycogen contents individuals and displayed the higher expressions in high glycogen content individuals. This might enhance the gluconeogenesis metabolism process by the transcriptionally regulating the expressions of phosphoenolpyruvate carboxykinase (PEPCK) and glucose 6-phosphatase (G6Pase). Finally, using genetic network analysis, we found several SAL that control both glycogen and several amino acids contents, which will provide important basis for their molecular breeding.

For GWAS analysis, the traits were usually controlled by many genes with small effects. Therefore, increasing the sample size will improve the power to recover meaningful associations [44]. Given this, selected the geographically distant accessions will maximize the genetic variance within the samples. Therefore, we have collected 427 individuals from different geographic regions. The same methods have been used in soybean [45], rice, pigs [46], etc., which could maximize the genetic variance. However, for the different environmental factors, collecting times and oyster ages, it cannot detect the phenotype directly with 427 individuals. In order to reduce the environmental effects for phenotype detection, we have conducted the common garden experiments and produced 427 half-sib families. For the F_1_ individuals in different families were cultured in the same marine environments and have the same male parent, their differences will reflect the genetic differentiation of their female parents. So, we have collected 30 individuals from each family, and their average phenotypic values were used to represent their female parents’ values. The same phenotypic detection method has been widely used in many other species [47–49], which indicate that this progeny-testing scheme used for the genetic evaluation of parents could reduce the environments noise which searching for GWAS. According to phenotype analysis, we found the largest change folds of glycogen and various amino acids contents ranged from 1.4- to 19.4, which was comparable with that of various crop varieties collected from different regions reported previously [40]. However, it must be illustrated that the sampling methods from different sites has the potential to introduce genetic heterogeneity, including local adaptation and genetic origin, leading to a non-causative marker being a better descriptor of the phenotype than a causative one [50].

Apart from genetic heterogeneity, another major challenge for GWAS analysis with our samples is population structure. It is well known that population structure generates associations between phenotypes and there need to do a statistical analysis of accounting for structure in GWAS. In our previous study, it was revealed that oyster could be divided into four populations based on their resequencing results [39]. In our results, we have added three abroad groups collected from Canada, Japan, and Korea. The structure analysis indicated that these three populations were different from those in China (Additional file 10: Figure S1), which was related with their geographical distribution pattern. The same result has been reported in several previous studies in mollusks, which was studied with several special polymorphism loci. Therefore, the population structure was used as a covariate within the GWAS model with the MLM model. Besides, LD decay revealed that oyster was one of the most diverse species with considerably rapid decay of LD when assessed at the genome-wide scale. The rapid decay of LD in oyster indicated a need for higher-density SNP panels for performing GWAS effectively. In our previous studies, it was revealed that 15× sequence depth was enough to obtain a sufficient number of accurately genotyped SNPs in oysters with a very high number of heterozygous sites [51]. However, 20× sequence depth will increase the coverage rate significantly. Therefore, in our results, we used the 20× sequence depth for resequencing, which was the first high depth resequencing for GWAS analysis of glycogen and amino acids contents. This also provide important references for other marine bivalves with the high polymorphisms for their GWAS analysis with genome resequencing.

Glycogen is the major source of glucose reserve stored in specific vesicular cells and is known to play a central role in providing energy for the maintenance and gametogenic development of bivalves [52]. The Pacific oyster *C. gigas* is rich in glycogen content, which is higher than that in other mollusks [53]. In previous study, several SNP loci have been identified for glycogen contents, including glycogen debranching enzyme, glycogen phosphorylase [20] and PPP1R3B [54]. Especially, PPP1R3B was identified based on our results, which promote glycogen synthesis together with protein phosphatase process. However, these results were mainly based on several candidate genes analysis. In the present study, we first conducted GWAS analysis with resequencing data and found the genome wide SNP loci associated with glycogen contents in molluscan. After filtering, a total of five genes regions located on three chromosomes were identified to be associated with glycogen content, which located on five scaffolds (scaffold1243, 1597, 340, 389 and 426). Further, four regions were also verified with the other wide populations with 288 individuals, which were most important candidate genomic regions for oyster breeding. We further conducted the gene function analysis. Further, for the high LD decay of SNP loci in these five genomic regions, we have identified 21 genes as the candidate genes. We mainly focused on the genes containing the non-synonymous mutations. We found that *CYP17A1* contained a non-synonymous SNP located in the enzyme active center and exhibited high expressions in individuals with higher glycogen content. CYP17A1 is a single gene-encoded protein that mediates 17α-hydroxylase and promotes gluconeogenesis by activating the transcriptional activity of the gluconeogenesis metabolism process in mammals [55]. In the present study, higher expression of gluconeogenesis genes (PEPCK and G6Pase) were also observed, which further indicated the stronger gluconeogenesis capacity of individuals with higher glycogen content. In previous studies, the mutations in CYP17A1 may result in the complete or partial loss of catalytic activities, inducing phenotypic variation in several species [56]. In the present study, according to the multiple alignment and three-dimensional protein structural modeling analysis, the non-synonymous SNP loci is located at a turn in β-sheet and formed the substrate-binding pocket. This mutation might affect the substrate binding activity of this enzyme. However, further studies should be conducted to analyze this genes function in glycogen metabolism process. Without functional validation, we could not determine whether these increases in glycogen content are caused by all these genes acting together as a cluster or by only one of these genes.

The aim of breeding is to aggregate many ideal traits into one variety, which required to understand the positive or negative correlation of these candidate traits [31]. Using GWAS analysis, we can explore the genetic regulation networks of different nutrient traits, which will help breeders to select effective markers to conduct molecular breeding. In many crops species, the genetic networks of different traits has been built, which were used for the breeding of many superior-quality crop varieties [31, 55]. However, in marine molluscan, the related studies have not been conducted until now. In this study, we have constructed the association networks across glycogen and many different amino acids. For example, we have identified three node SNP loci, which were all related with glycogen, protein, and Asp content in oysters. These polymorphism information was very important for oyster breeding of these nutrient traits. The node SNP loci will be used for breeding of these heritable covariation traits in on variety; however the specific SAL will be used for breeding of the specific trait.

## Conclusions

In summary, the present study provides a large dataset of loci and genes responsible for important traits in oyster, such as glycogen, protein, and amino acids, which will facilitate future functional studies and breeding development. Furthermore, we have constructed association networks among different traits and identified some SALs that function as key nodes connecting different traits, but most SALs specifically regulated individual traits. This information will guide breeders attempting to establish a clear strategy for genetic improvement. However, the genes associated with nutrient traits were not involved in their traditional key metabolism pathways, indicating the existence of a new regulation mechanism of glycogen, protein, and amino acid metabolism processes in oysters. Further work will be necessary to verified the functional mechanism of these specific genes underlying these breeding target traits.

## Methods

### Sampling and phenotyping for GWAS analysis

For the GWAS, oysters (*C. gigas*) from 26 different environmental conditions were collected during the summer of 2013. After culturing for approximately 1 month in farms, the gill of 427 females were collected and used for resequencing. At the same time, these females were mated with one male cultured in Qingdao, generating 427 half-sub families. These 427 families were cultured in the same environment for approximately 1 year, and then 30 F_1_ individuals from each family line were collected and used for phenotype measurement [11]. For these F_1_ individuals have the same male parent and the same culturing environments, their phenotypic differentiation may be induced by the female parent’s differentiation. The same methods have been widely used in many other species [47–49]. The breeding, phenotype and genotype detection methods have been described in Additional file 10: Figure S1.

The content of glycogen and protein was analyzed by near infrared reflectance spectroscopy as previously described [56], with minor modifications. The amino acid content was detected following the method of Mao et al. (2015) using the automatic amino-acid analyzer (Eppendorf LC-3000, Hamburg, Germany) [42]. The phenotypic correlation matrix was obtained by Pearson product-moment correlation coefficients using the “corrplot” package in R.

### DNA isolation and genome sequencing

The genomic DNA of 427 individuals from 26 locations was extracted from the gills by the phenol-chloroform method. The DNA (1–15 μg) was fragmented using the Covaris system (Inc., Woburn, MA) to obtain 200–800 bp fragments. The DNA fragments were then treated according to the Illumina DNA sample preparation protocol. The fragments were end-repaired, A-tailed, ligated to paired-end adaptors, and PCR amplified with 500-bp inserts for library construction. Sequencing was performed to generate 100-bp paired-end reads on the HiSeq 2000 platform (Illumina) according to the manufacture’s standard protocol.

### Sequence alignment, SNP calling, and population structure analysis

The SNP calling and population structure analysis were conducted as reported previously [33]. The Burrows-Wheeler Aligner (BWA) software was used for alignment with the reference genome (GenBank accession No., GCA_000297895.1) (bwa mem –M -t 10 -T 20) [43]. The GATK [57] module Haplotype Caller and SAMtools [58] were used for variant calling and the concordance variants were filtered with the parameter “QD < 2.0 || FS > 30.0 || MQ < 40.0 || DP < 6 || DP > 888 || ReadPosRankSum < -8.0 || BaseQRankSum < -8”. Further, the INDELs were filtered with “QD < 2.0 || FS > 30.0 || ReadPosRankSum < -8.0”. The variants were used for population variant calling using the GATK Haplotype Caller module and the parameter was set as “--genotyping_mode DISCOVERY -stand_emit_conf 10 -stand_call_conf 30”.

The phylogenetic tree was constructed with TreeBeST (http://treesoft.sourceforge.net/treebest.shtml) using the SNPs at a population scale. Furthermore, the Discriminant Analysis of Principal Components (DAPC) analysis was performed using the R package.

### Genome-wide association study

To minimize false positives and increase statistical value, population structure and cryptic relationships were considered. The compressed mixed linear model program GAPIT was used for the association analysis [59]. The first three PCA values, which were derived from whole-genome SNPs, were used as fixed effects in the mixed model to correct stratification. Random effect was estimated from the groups clustered based on the kinship among all accessions. Only the SNPs with MAF ≥ 0.05 and missing rate < 0.1 in the population were used in the GWAS. We defined the whole-genome significance cutoff as Bonferroni test threshold. For SNP GWAS, the threshold was set as *P*-value < 10^− 6^ [60]. The candidate genes were searched between the most significant SNP loci within 100 kb in a scaffold.

### GWAS results validation using wild 288 individuals

In order to validate the GWAS results, we have conducted association analysis using 288 wild one-year-old oysters collected from the Jiao Nan Aquaculture Farm in Qingdao, in May 2014. These juvenile oysters were sampled in the wild and then reared under common conditions. They were genotyped using a 190 K SNP chip, which was previously described in [41]. The contents of glycogen, protein, and amino acids were also determined by the above-mentioned methods. The association analysis was also performed by GAPID.

### Functional analysis of candidate genes in the GWAS associated loci

We used the following strategy to narrow down candidate genes. First, according to the GWAS associated loci, we estimated the candidate region by pairwise LD correlation. Second, based on our assembled genome sequence for *C. gigas*, we analyzed the SNP types located in the candidate region. We focused on the genes with associated non-synonymous SNPs significantly associated with the traits in the GWAS and those that could induce changes in amino acids. Third, we checked whether these candidate genes with associated non-synonymous SNPs had different expression patterns in high and low phenotype individuals by the real time polymerase chain reaction (PCR). Finally, we conducted gene-based analysis (GBA) with PCR-based sequencing to confirm the associated non-synonymous SNPs. We also conducted the association analysis with SHEsis Plus Online Version-Beta (http://analysis.bio-x.cn/) and classified the samples into distinct haplotypes.

### Association network construction

The software Cytoscape was used to construct association networks [61]. The traits with their corresponding SAL were treated as nodes, and the links between the trait and SAL, and between the SAL and SAL were treated as edges. The link between two SALs was represented by their average LD (inter-LD) as follows [31]:

$$ \mathrm{Inter}\hbox{-} \mathrm{LD}=\frac{1}{2}\times \frac{\mathrm{LD}\left(\mathrm{SAL}1,\mathrm{SAL}2\right)}{\mathrm{PmaxLD}\left(\mathrm{SAL}1\right)}+\frac{\mathrm{LD}\left(\mathrm{SAL}1,\mathrm{SAL}2\right)}{\mathrm{PmaxLD}\left(\mathrm{SAL}2\right)} $$

where, LD (SAL1, SAL2) is the pairwise LD value (*r*^2^) between all SNPs from SAL1 region to all SNPs from SAL2 region; PmaxLD (SAL1) or PmaxLD (SAL2) are the largest possible LD value within the SAL1 or SAL2 region, respectively, obtained by calculating the mean *r*^2^ of each SNP to all the SNPs from the SAL1 or SAL2 region and choosing the maximum mean LD to represent the region’s PmaxLD; Pairwise *r*^2^ values represent all significant SNPs. Only the average *r*^2^ ≥ 0.1 were selected to draw the networks.

### Real-time PCR

Among the 288 individuals, the top 15 oysters with extreme high and low glycogen or amino acid content were selected for the real-time PCR. Forty oysters were collected for RNA extraction. The total RNA was isolated using the Trizol reagent (Invitrogen). The RNA yield and purity were determined spectrophotometrically (BioPhotometer; Eppendorf, Hamburg, Germany) at 260 and 280 nm. The RNA integrity was assessed by 1.2% agarose gel electrophoresis.

For the quantitative real time PCR (q-PCR), the RNA sample was reverse transcribed using a cDNA synthesis kit (DRR420; Takara) and qRT-PCR was performed using the ABI7500 fast Real-Time Detection System (Applied Biosystems, USA). The elongation factor (*EF*) gene was chosen as the internal standard. The qRT-PCR was carried out in triplicate with a reaction mixture of total volume 20 μL containing 10 μL of SYBR Green 2X Supermix (Takara), 1 μL of 1:100 diluted cDNA, 0.4 μL each of the forward and reverse primers, 0.4 μL of ROX Dye II, and 7.8 μL of DEPC H_2_O. The PCR involved two steps: 95 °C for 30 s, followed by 40 cycles at 95 °C for 3 s and 60 °C for 30 s. The analysis was based on the Ct values of the PCR products. Melting curve analysis of the products was performed at the end of each PCR amplification. The Ct values for the comparison between the amplified genes and *EF* gene (ΔCt) were calculated. The blank group was used as the reference sample (i.e., as calibrator). The ΔCt of each sample was then subtracted from the ΔCt of the calibrator, and this difference is called the ΔΔCt value. The expression level of the target genes was then calculated as 2^-ΔΔCt^.

## Additional files

Additional file 1: ** Table S1** Sample information of Pacific oysters used in this study. (XLSX 10 kb)

Additional file 2: ** Table S2** Summary of the results of whole-genome resequencing. (XLSX 11 kb)

Additional file 3: ** Table S3** Four hundred and twenty-seven oysters collected from the world-wide scale and their phenotyping values, including the content of glycogen, protein, and 17 different amino acids. (XLSX 66 kb)

Additional file 4: ** Table S4** Fold changes of different nutrient quality traits in 426 individuals. (XLSX 12 kb)

Additional file 5: ** Table S5** Correlation analysis among different traits. Upper matrix indicates correlation value and lower matrix indicates significance (**P < 0.01, *P < 0.05). (XLSX 14 kb)

Additional file 6: ** Table S6** Genome-wide significant association signals of quality traits in *C. gigas* using the compressed MLM model. (XLSX 65 kb)

Additional file 7: ** Table S7** The significant SNP loci of glycogen contents with the genome-wide analysis for 288 individuals using the compressed MLM model. (XLSX 243 kb)

Additional file 8: ** Table S8** The significant SNP loci of protein contents for the genome-wide analysis with 288 individuals using the compressed MLM model. (XLSX 250 kb)

Additional file 9: ** Table S9** Link loci across different traits. (XLSX 33 kb)

Additional file 10: ** Figure S1** Phylogenetic tree analysis of Pacific oysters using the whole-genome SNPs. *C. angulata* was used as the outgroup. (DOCX 141 kb)

Additional file 11: ** Figure S2** F_1_ breeding methods of 427 individuals collected from the world-wide scale. The females collected were mated with the males cultured in Qingdao and generated 427 family lines. They were cultured in the same environment for approximately one year, and then 30 individuals in each family line were collected and used for phenotype measurement. (DOCX 124 kb)

Additional file 12: ** Figure S3** Histogram of glycogen and amino acids content in oysters. All quality traits presented normal distribution, except for Met and Cys. X axis represents the value of different traits, whereas, the y axis represents number of individuals. (DOCX 235 kb)

Additional file 13: ** Figure S4** Amino acid composition in all 427 individuals. (A) Among all detected amino acids. Glu and Tau accounted for 14 and 11% of TAA content, respectively, and they represent the amino acids with the highest content in *C. gigas*. (B) Among all detected amino acids, six DAA accounted for 41% of TAA content. (DOCX 692 kb)

Additional file 14: ** Figure S5** Genome-wide analysis of glycogen, protein, and amino acids components. The left panel shows the Manhattan plots of the MLM model. The X axis shows the genomic position in 10 chromosomes and the Y axis shows the significance expressed as -log_10_-transformed *P*-value. The right panel shows the Quantile-quantile plot of the MLM model. (DOCX 2787 kb)

Additional file 15: ** Figure S6** Pairwise LD analysis of the 100 kb-region on both sides of the leading SNP of glycogen and protein. The upper panel shows the GWAS results of the 100 kb-region on both sides of the leading SNP of the trait, whereas, the panel below shows the pairwise LD analysis of the SNPs (*P* < 2 × 10^− 4^) included in the region. (DOCX 4156 kb)

Additional file 16: ** Figure S7** Pairwise LD analysis of the 100 kb-region on both sides of the leading SNP of amino acids. The upper panel shows the GWAS results of the 100 kb-region on both sides of the leading SNP of the trait, whereas, the panel below shows the pairwise LD analysis of the SNPs (*P* < 2 × 10^− 4^) included in the region. (DOCX 2658 kb)

Additional file 17: ** Figure S8** Indidate gene identification. First part shows the Manhattan plots of traits. Red horizontal dashed line indicates the genome-wide significance threshold. Second part presents 0.1-Mb region on each side of the peak SNP, the position of which is indicated by a vertical red line with red triangle. Bottom of each panel shows the annotated genes of the 200-kb region. Gene is indicated by black boxes. If the significant SNP was located in the gene, the gene name is highlighted in red bold font, and the coding region of the gene is indicated by black vertical lines. (DOCX 220 kb)

Additional file 18: ** Figure S9** Predicted 3D structure of CYP17A1 proteins of different genotypes with SWISS-MODEL Workspace (https://swissmodel.expasy.org/). (DOCX 1099 kb)

Additional file 19: ** Figure S10** Genome-wide association study of Asp (A), His (B), Leu (C), Cys (D), and Met (E) content. The first section of each panel shows the Manhattan plot of traits. The negative log10-transformed *P*-values from the genome-wide scan are plotted against position on each of the 10 chromosomes. Red horizontal dashed line indicates the genome-wide significance threshold. Second part exhibits the 0.1-Mb region on each side of the peak SNP and its position is indicated by a vertical red line with red triangle. The bottom of each panel shows the annotated genes of the 200-kb region. Significant SNP that surpassed the threshold is indicated by red plots and peak SNP is indicated by purple plots. The dotted lines with arrows represent the traits related to the peak SNP. Gene is indicated by black boxes. If the significant SNP was located in the gene, the gene name is highlighted in red bold font and the coding region of the gene was indicated by black vertical lines. (DOCX 768 kb)

## Data Availability

The Whole genome resequencing and transcriptomes data sets were deposited in the Sequence Read Archive (SRA) database under the accession numbers PRJNA394055.

## Abbreviations

CYP17A1: Cytochrome P450 17A1
G6Pase: Glucose 6-phosphatase
GWAS: Genome wide association analysis
LD: Linkage disequilibrium
NJ: Neighbor-joining tree
PEPCK: Phosphoenolpyruvate carboxykinase
PLC: Perlucin
SAL: Significantly associated loci
SNP: Single nucleotide polymorphism
TAA: Total amino acid content
