# Supplementary figures and images for: Genome-wide association analysis of nutrient traits in the oyster Crassostrea gigas: genetic effect and interaction network
Source: BMC Genomics. 2019 Jul 31;20:625. doi: 10.1186/s12864-019-5971-z (PMC6670154; doi:10.1186/s12864-019-5971-z)

**
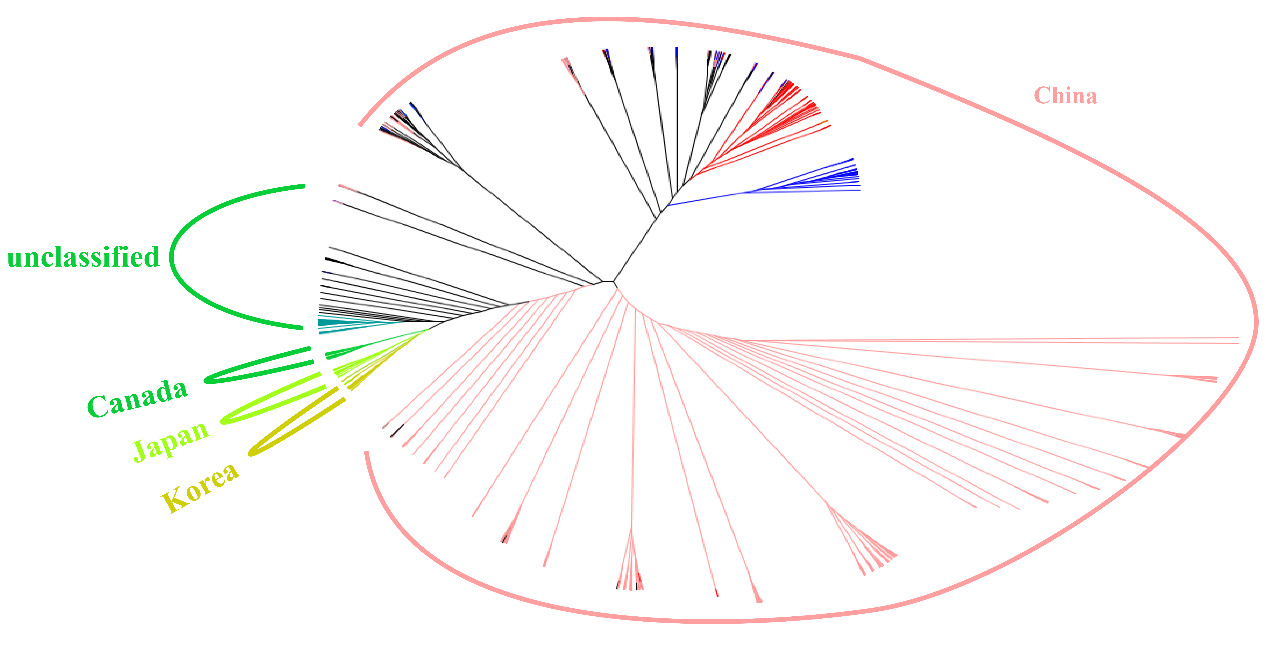
**

**Fig. S1** Phylogenetic tree analysis of Pacific oysters using the whole-genome SNPs.

Supplement: Supplementary file 10 — Figure S1 Phylogenetic tree analysis of Pacific oysters using the whole-genome SNPs. C. angulata was used as the outgroup. (DOCX 141 kb) [file 12864_2019_5971_MOESM10_ESM.docx]
