# Supplementary material for: Genome-wide association analysis of nutrient traits in the oyster Crassostrea gigas: genetic effect and interaction network
Source: BMC Genomics. 2019 Jul 31;20:625. doi: 10.1186/s12864-019-5971-z (PMC6670154; doi:10.1186/s12864-019-5971-z)
Supplement: Supplementary file 11 — Figure S2 F1 breeding methods of 427 individuals collected from the world-wide scale. The females collected were mated with the males cultured in Qingdao and generated 427 family lines. They were cultured in the same environment for approximately one year, and then 30 individuals in each family line were collected and used for phenotype measurement. (DOCX 124 kb) [file 12864_2019_5971_MOESM11_ESM.docx]

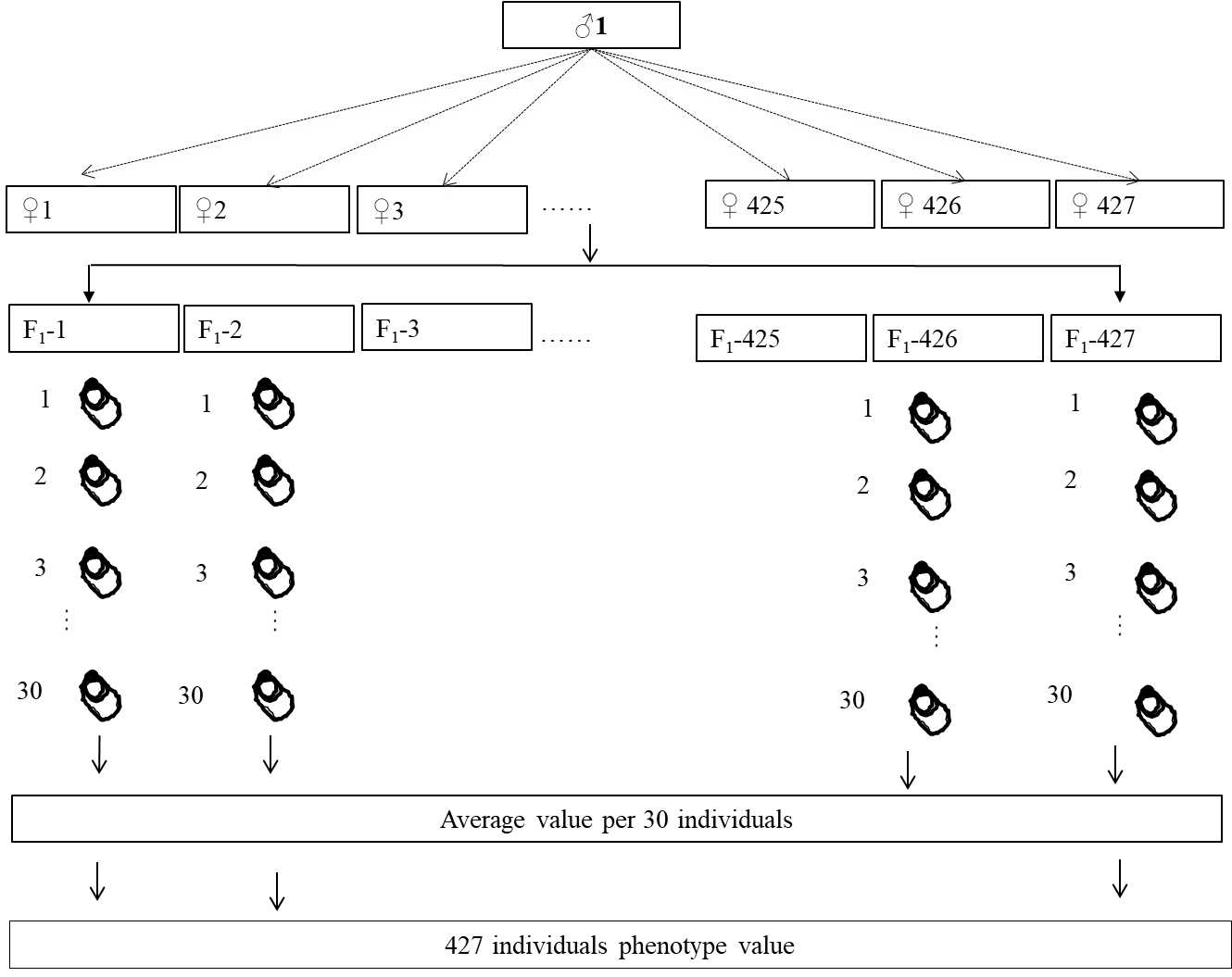


**Fig. S2** F_1_ breeding methods of 427 individuals collected from the world-wide scale. The females collected were mated with the males cultured in Qingdao and generated 427 family lines. They were cultured in the same environment for approximately one year, and then 30 individuals in each family line were collected and used for phenotype measurement.
