# Supplementary material for: Genome-wide association analysis of nutrient traits in the oyster Crassostrea gigas: genetic effect and interaction network
Source: BMC Genomics. 2019 Jul 31;20:625. doi: 10.1186/s12864-019-5971-z (PMC6670154; doi:10.1186/s12864-019-5971-z)
Supplement: Supplementary file 12 — Figure S3 Histogram of glycogen and amino acids content in oysters. All quality traits presented normal distribution, except for Met and Cys. X axis represents the value of different traits, whereas, the y axis represents number of individuals. (DOCX 235 kb) [file 12864_2019_5971_MOESM12_ESM.docx]

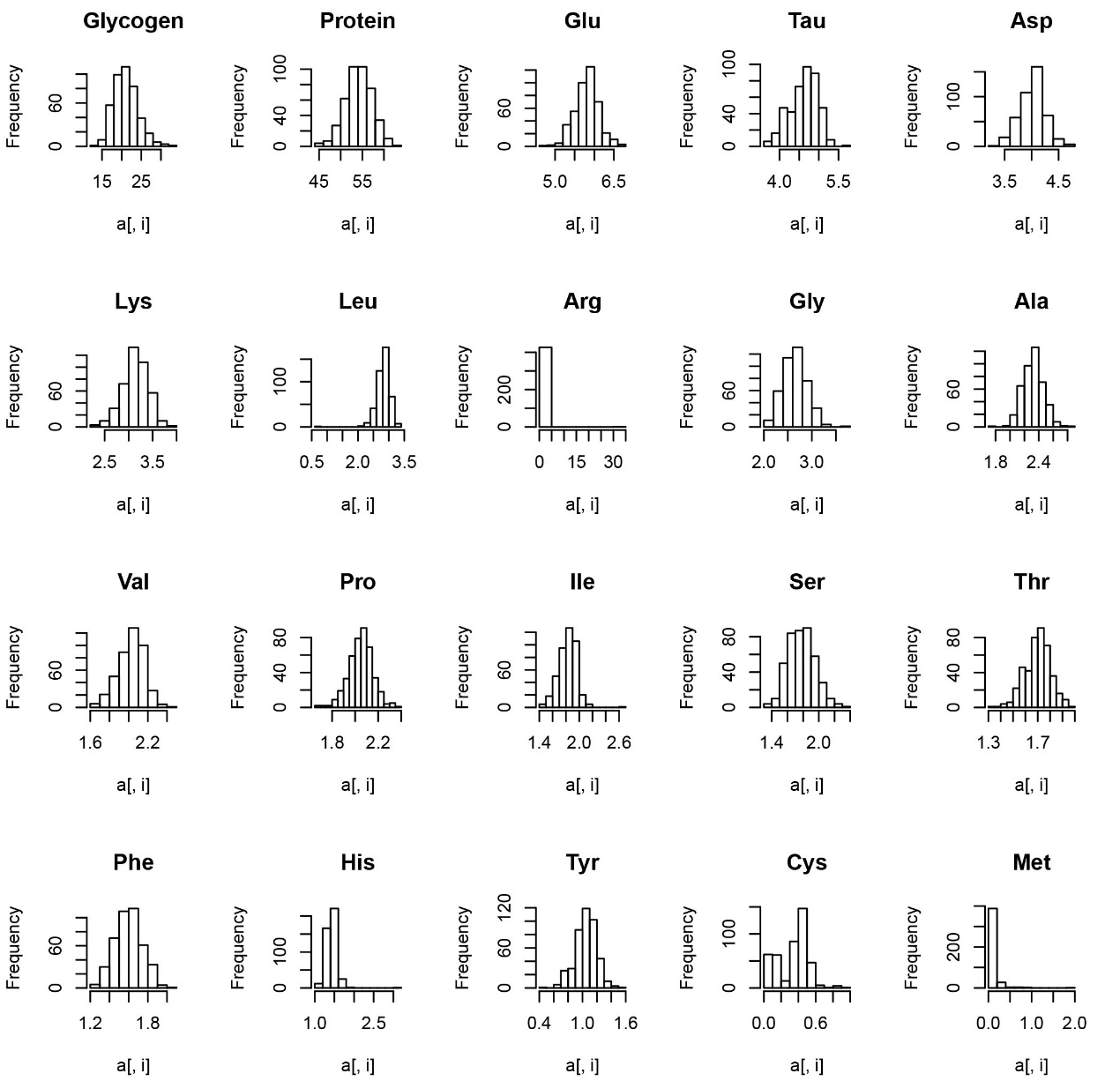


**Fig.** **S3** Histogram of glycogen and amino acids content in oysters. All quality traits presented normal distribution, except for Met and Cys. X axis represents the value of different traits, whereas, the y axis represents number of individuals.
