# Supplementary material for: Genome-wide association analysis of nutrient traits in the oyster Crassostrea gigas: genetic effect and interaction network
Source: BMC Genomics. 2019 Jul 31;20:625. doi: 10.1186/s12864-019-5971-z (PMC6670154; doi:10.1186/s12864-019-5971-z)
Supplement: Supplementary file 13 — Figure S4 Amino acid composition in all 427 individuals. (A) Among all detected amino acids. Glu and Tau accounted for 14 and 11% of TAA content, respectively, and they represent the amino acids with the highest content in C. gigas. (B) Among all detected amino acids, six DAA accounted for 41% of TAA content. (DOCX 692 kb) [file 12864_2019_5971_MOESM13_ESM.docx]

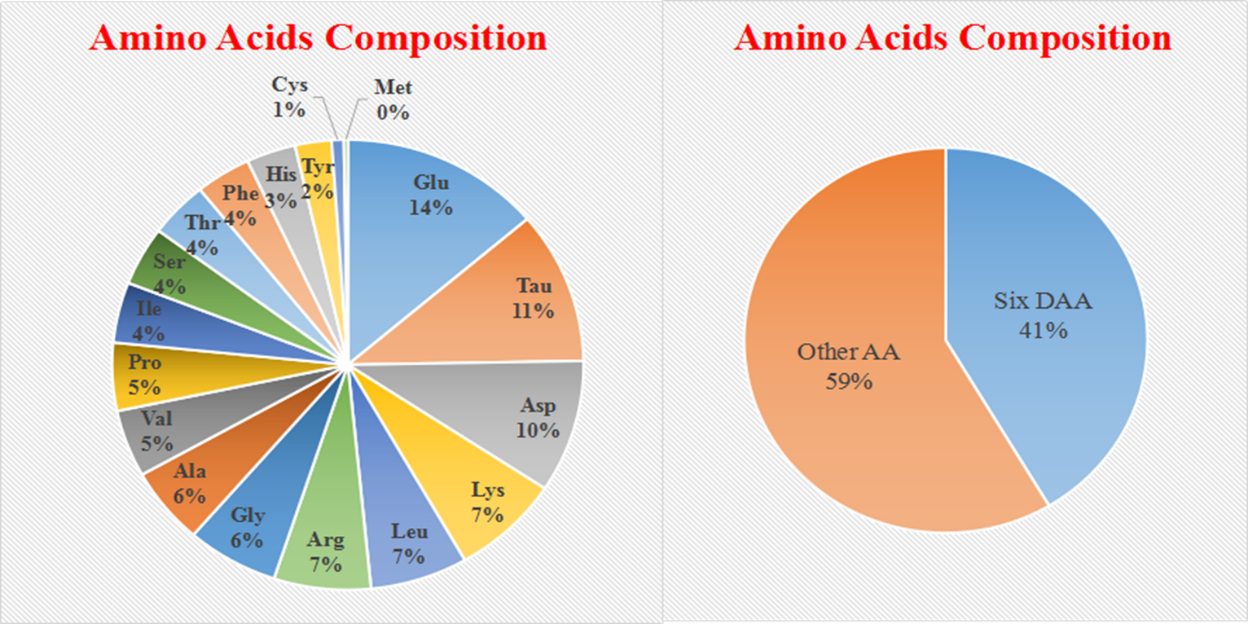


**Fig. S4 Amino acid composition in all 427 individuals.** (A) Among all detected amino acids. Glu and Tau accounted for 14% and 11% of TAA content, respectively, and they represent the amino acids with the highest content in *C. gigas*. (B) Among all detected amino acids, six DAA accounted for 41% of TAA content.
