# Supplementary material for: Genome-wide association analysis of nutrient traits in the oyster Crassostrea gigas: genetic effect and interaction network
Source: BMC Genomics. 2019 Jul 31;20:625. doi: 10.1186/s12864-019-5971-z (PMC6670154; doi:10.1186/s12864-019-5971-z)
Supplement: Supplementary file 14 — Figure S5 Genome-wide analysis of glycogen, protein, and amino acids components. The left panel shows the Manhattan plots of the MLM model. The X axis shows the genomic position in 10 chromosomes and the Y axis shows the significance expressed as -log10-transformed P-value. The right panel shows the Quantile-quantile plot of the MLM model. (DOCX 2787 kb) [file 12864_2019_5971_MOESM14_ESM.docx]

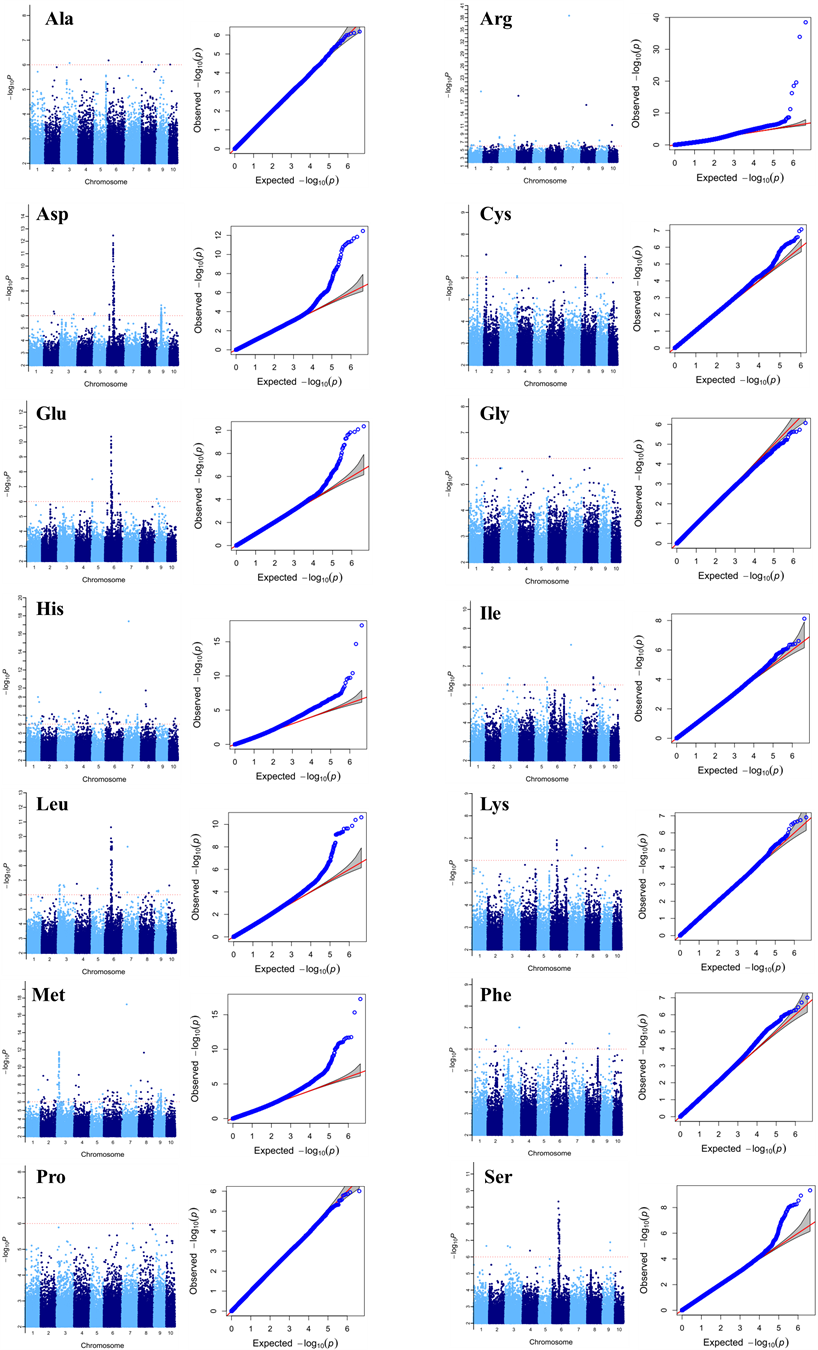


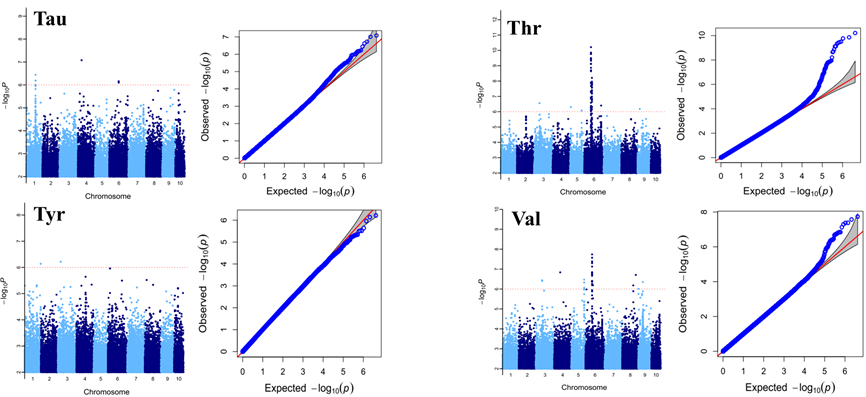


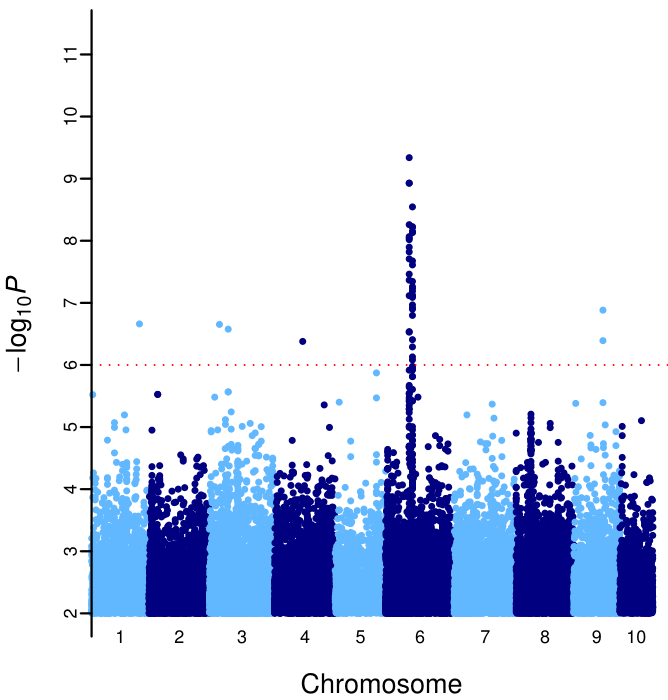

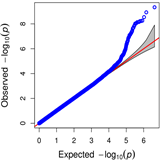


**Ser**


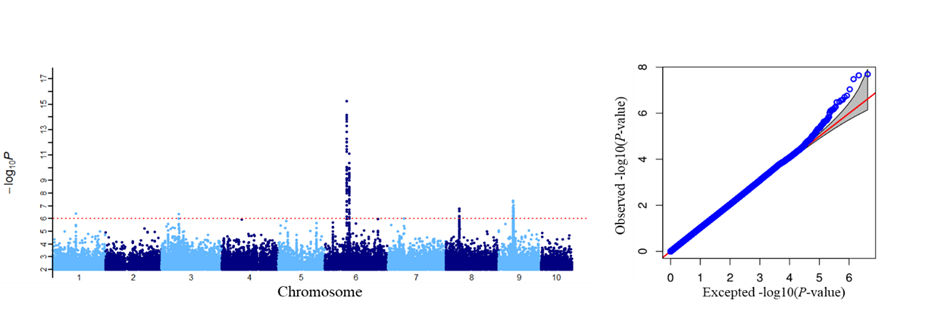

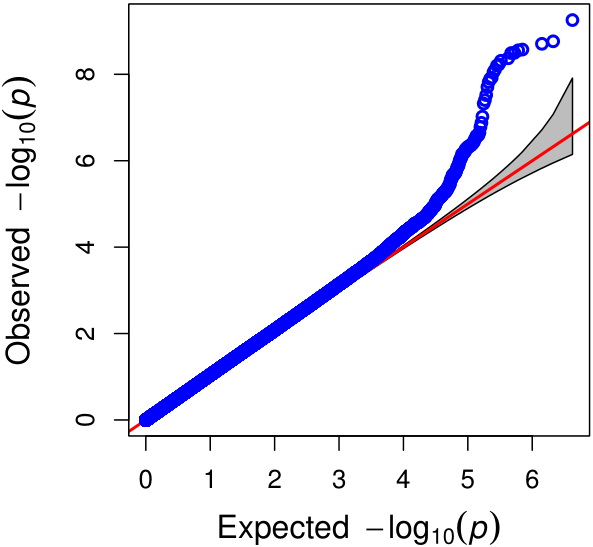


**Protein**

**Glycogen**


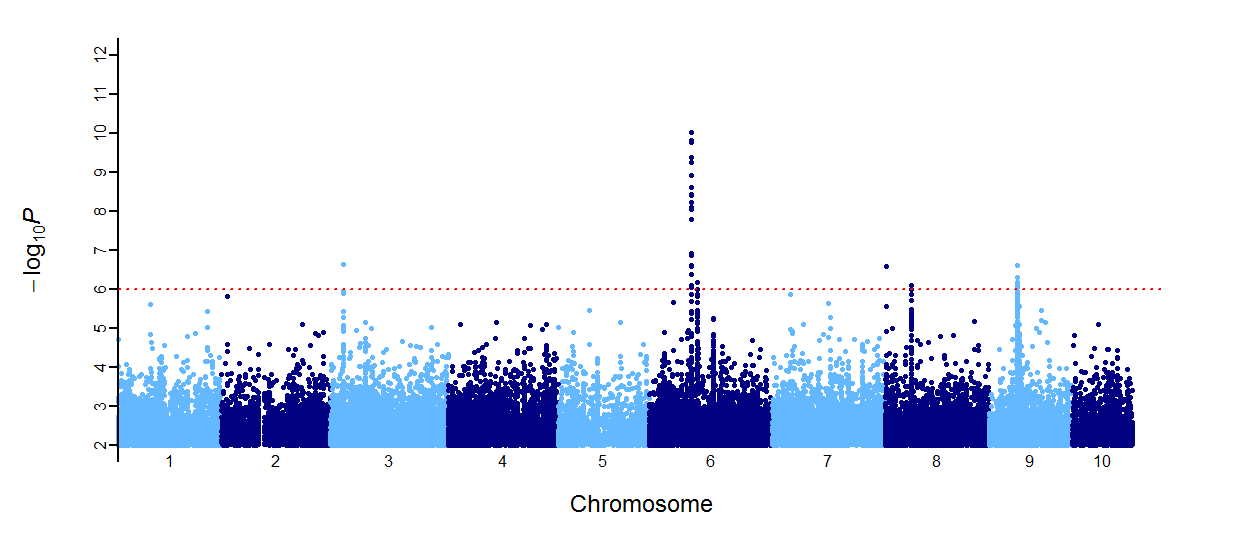

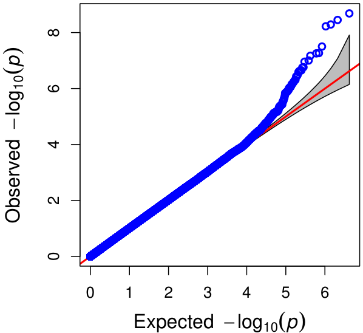


**Fig. S5 Genome-wide analysis of glycogen, protein, and amino acids components.** The left panel shows the Manhattan plots of the MLM model. The X axis shows the genomic position in 10 chromosomes and the Y axis shows the significance expressed as -log_10_-transformed *P*-value. The right panel shows the Quantile-quantile plot of the MLM model.
