# Supplementary material for: Genome-wide association analysis of nutrient traits in the oyster Crassostrea gigas: genetic effect and interaction network
Source: BMC Genomics. 2019 Jul 31;20:625. doi: 10.1186/s12864-019-5971-z (PMC6670154; doi:10.1186/s12864-019-5971-z)
Supplement: Supplementary file 16 — Figure S7 Pairwise LD analysis of the 100 kb-region on both sides of the leading SNP of amino acids. The upper panel shows the GWAS results of the 100 kb-region on both sides of the leading SNP of the trait, whereas, the panel below shows the pairwise LD analysis of the SNPs (P < 2 × 10− 4) included in the region. (DOCX 2658 kb) [file 12864_2019_5971_MOESM16_ESM.docx]

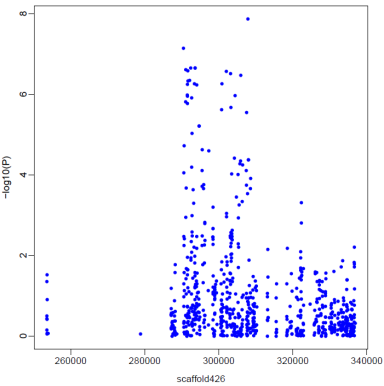


Glu


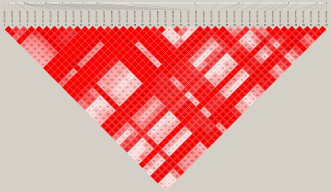

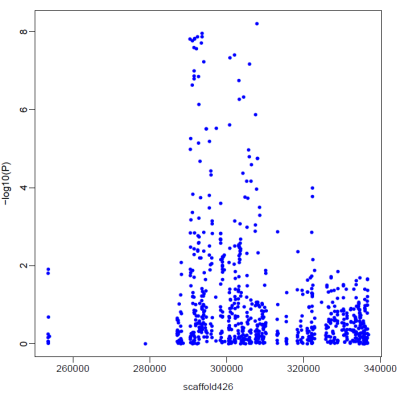


Thr


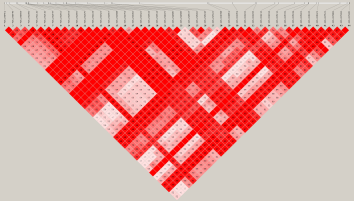

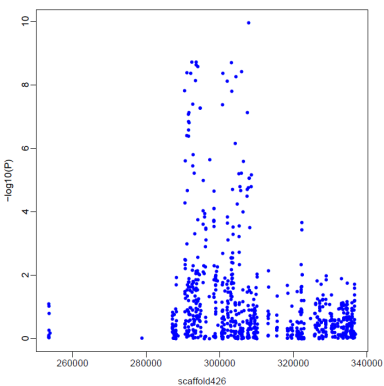


Asp


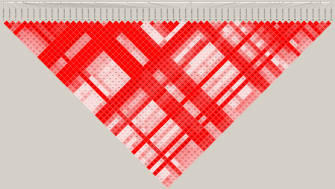

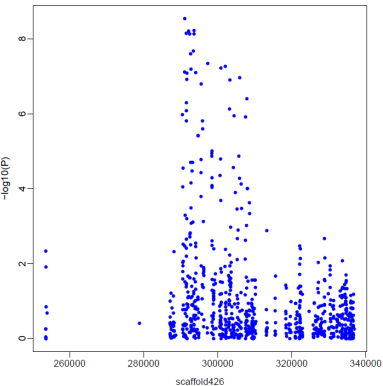


Ser


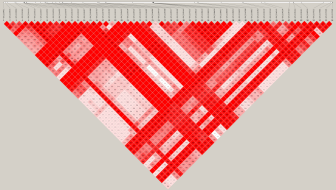

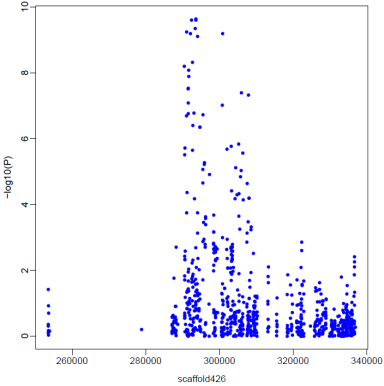


Leu


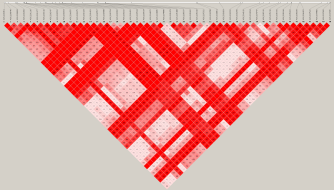

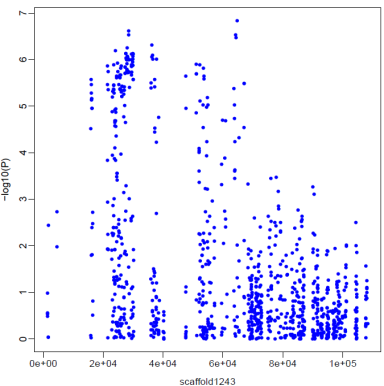


Asp


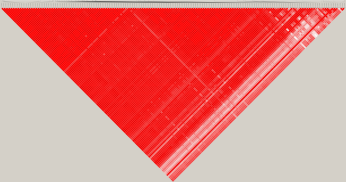

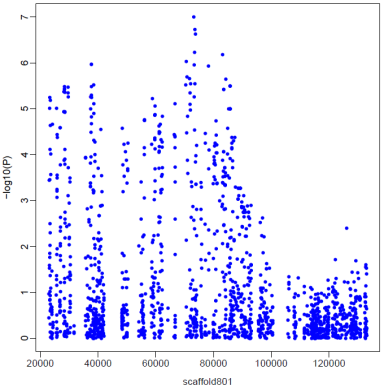


Tau


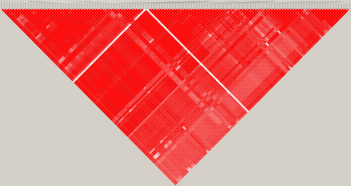

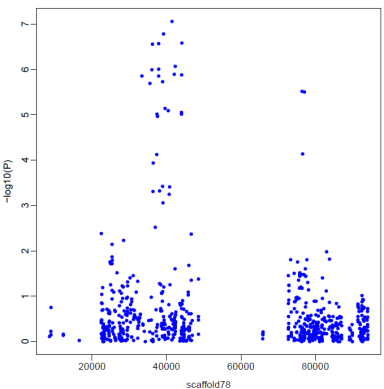


His


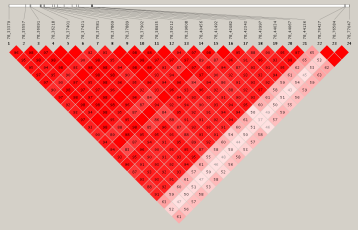


**Fig. S7 Pairwise LD analysis of the 100kb-region on both sides of the leading SNP of amino acids.** The upper panel shows the GWAS results of the 100 kb-region on both sides of the leading SNP of the trait, whereas, the panel below shows the pairwise LD analysis of the SNPs (P < 2 × 10^-4^) included in the region.
