# Supplementary material for: Genome-wide association analysis of nutrient traits in the oyster Crassostrea gigas: genetic effect and interaction network
Source: BMC Genomics. 2019 Jul 31;20:625. doi: 10.1186/s12864-019-5971-z (PMC6670154; doi:10.1186/s12864-019-5971-z)
Supplement: Supplementary file 17 — Figure S8 Indidate gene identification. First part shows the Manhattan plots of traits. Red horizontal dashed line indicates the genome-wide significance threshold. Second part presents 0.1-Mb region on each side of the peak SNP, the position of which is indicated by a vertical red line with red triangle. Bottom of each panel shows the annotated genes of the 200-kb region. Gene is indicated by black boxes. If the significant SNP was located in the gene, the gene name is highlighted in red bold font, and the coding region of the gene is indicated by black vertical lines. (DOCX 220 kb) [file 12864_2019_5971_MOESM17_ESM.docx]

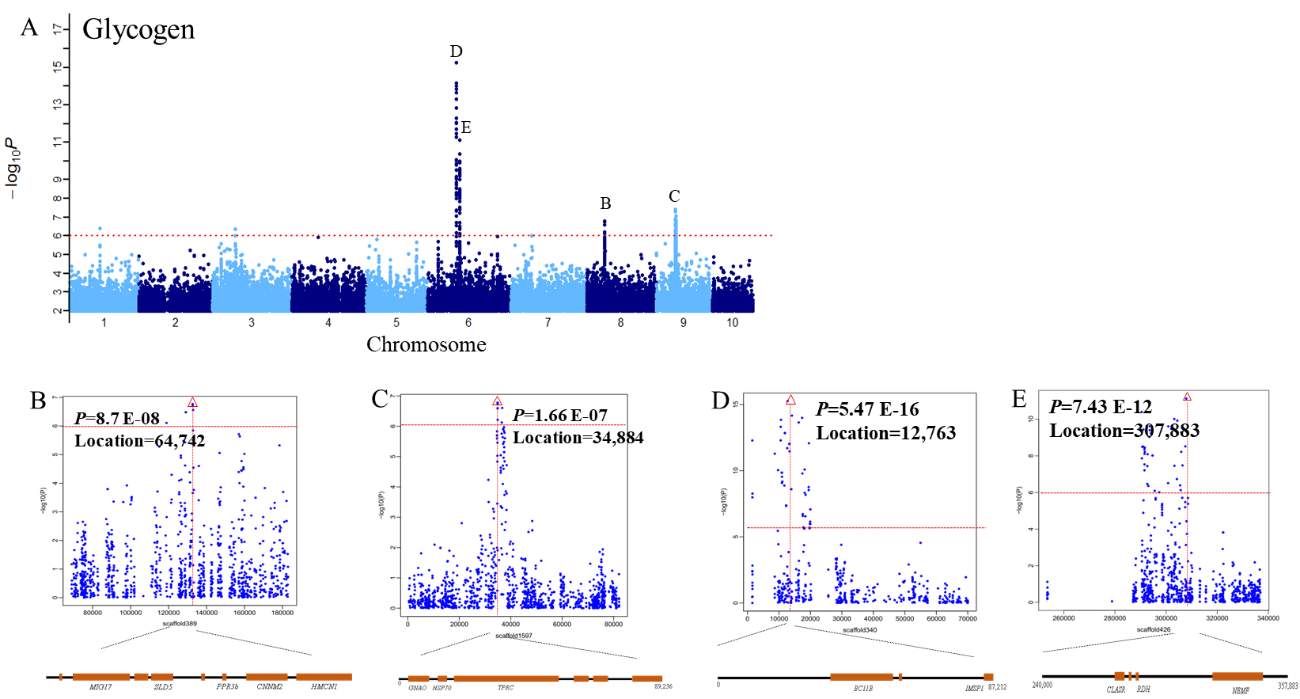


**Fig. S8** **Genome-wide association study of glycogen and candidate gene identification.** First part shows the Manhattan plots of traits. Red horizontal dashed line indicates the genome-wide significance threshold. Second part presents 0.1-Mb region on each side of the peak SNP, the position of which is indicated by a vertical red line with red triangle. Bottom of each panel shows the annotated genes of the 200-kb region. Gene is indicated by black boxes. If the significant SNP was located in the gene, the gene name is highlighted in red bold font, and the coding region of the gene is indicated by black vertical lines.
