# Supplementary material for: Genome-wide association analysis of nutrient traits in the oyster Crassostrea gigas: genetic effect and interaction network
Source: BMC Genomics. 2019 Jul 31;20:625. doi: 10.1186/s12864-019-5971-z (PMC6670154; doi:10.1186/s12864-019-5971-z)
Supplement: Supplementary file 18 — Figure S9 Predicted 3D structure of CYP17A1 proteins of different genotypes with SWISS-MODEL Workspace (https://swissmodel.expasy.org/). (DOCX 1099 kb) [file 12864_2019_5971_MOESM18_ESM.docx]

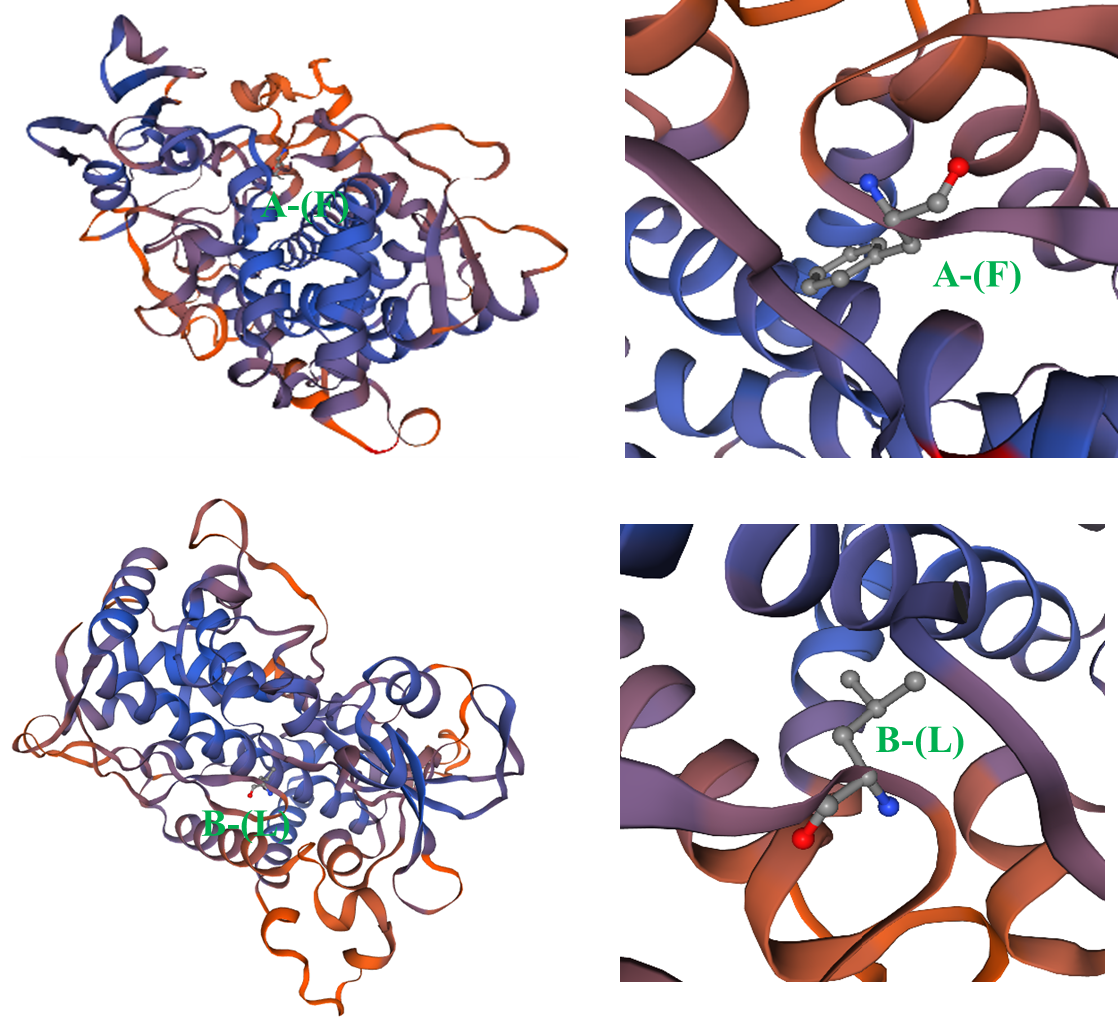


**Fig. S9** Predicted 3D structure of CYP17A1 proteins of different genotypes with SWISS-MODEL Workspace (<https://swissmodel.expasy.org/>).
