# Supplementary material for: Genome-wide association analysis of nutrient traits in the oyster Crassostrea gigas: genetic effect and interaction network
Source: BMC Genomics. 2019 Jul 31;20:625. doi: 10.1186/s12864-019-5971-z (PMC6670154; doi:10.1186/s12864-019-5971-z)
Supplement: Supplementary file 19 — Figure S10 Genome-wide association study of Asp (A), His (B), Leu (C), Cys (D), and Met (E) content. The first section of each panel shows the Manhattan plot of traits. The negative log10-transformed P-values from the genome-wide scan are plotted against position on each of the 10 chromosomes. Red horizontal dashed line indicates the genome-wide significance threshold. Second part exhibits the 0.1-Mb region on each side of the peak SNP and its position is indicated by a vertical red line with red triangle. The bottom of each panel shows the annotated genes of the 200-kb region. Significant SNP that surpassed the threshold is indicated by red plots and peak SNP is indicated by purple plots. The dotted lines with arrows represent the traits related to the peak SNP. Gene is indicated by black boxes. If the significant SNP was located in the gene, the gene name is highlighted in red bold font and the coding region of the gene was indicated by black vertical lines. (DOCX 768 kb) [file 12864_2019_5971_MOESM19_ESM.docx]

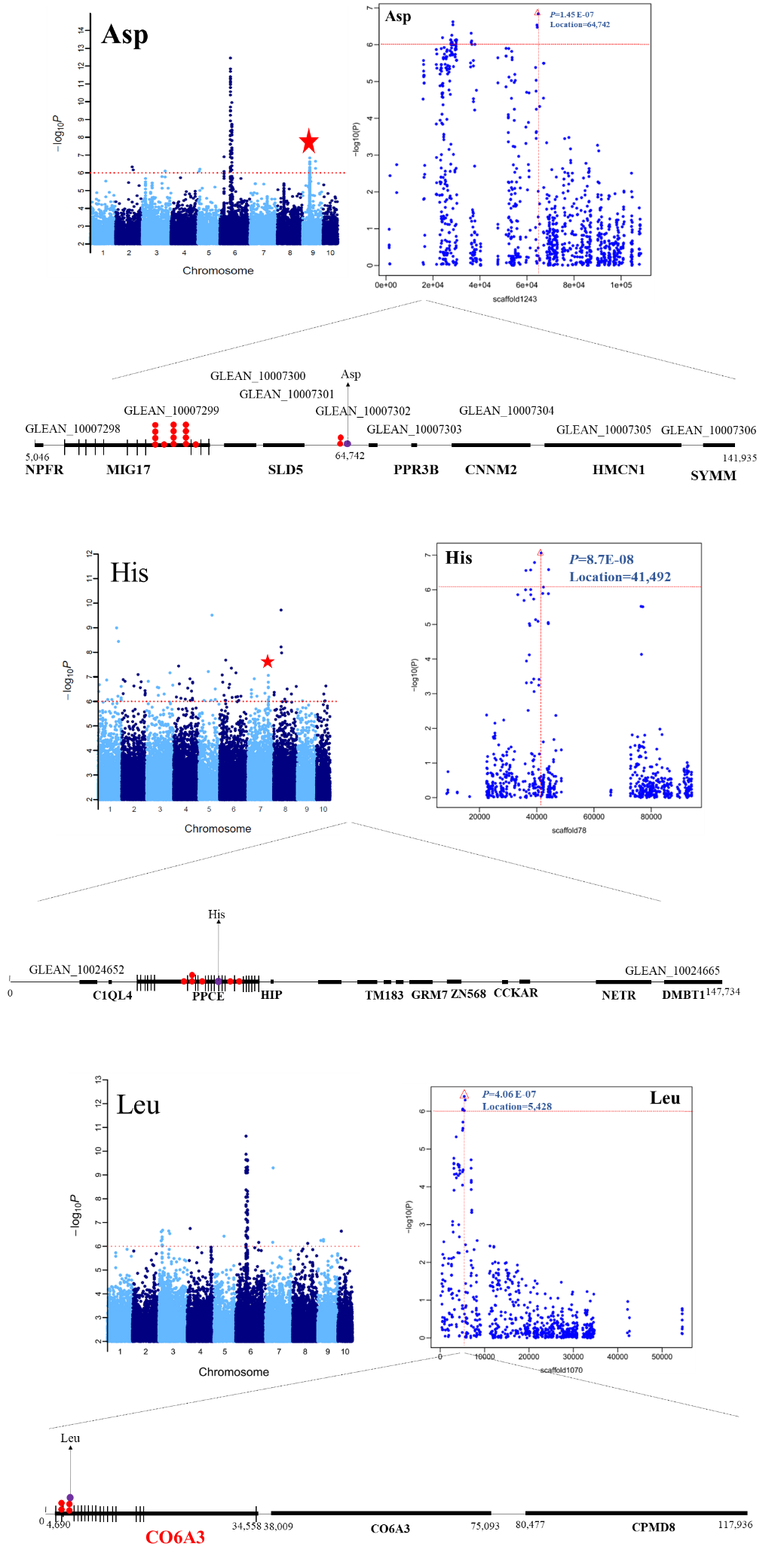


**C**

**B**

**A**


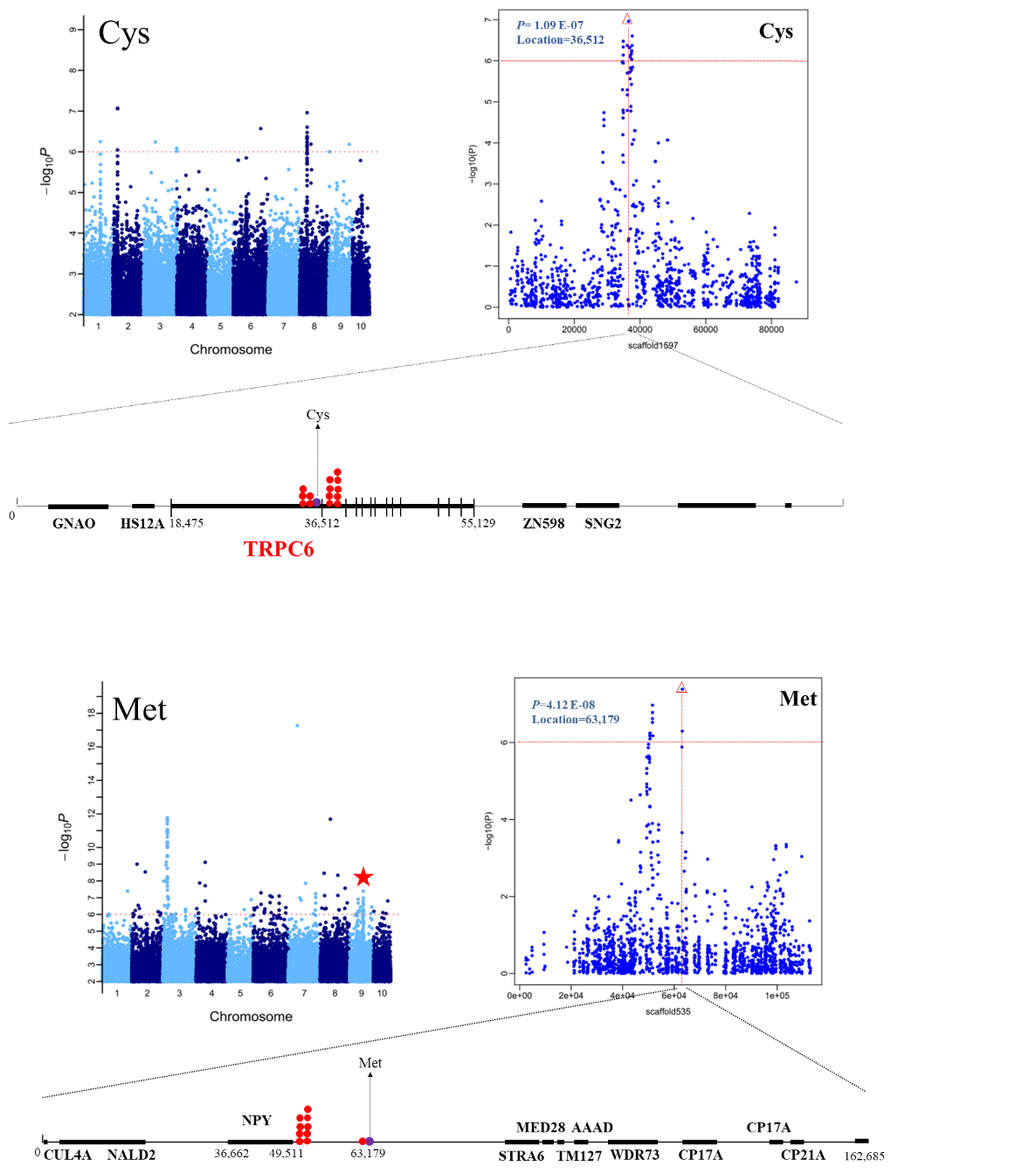


**E**

**D**

**Fig. S10 Genome-wide association study of Asp (A), His (B), Leu (C), Cys (D), and Met (E) content.** The first section of each panel shows the Manhattan plot of traits. The negative log10-transformed *P*-values from the genome-wide scan are plotted against position on each of the 10 chromosomes. Red horizontal dashed line indicates the genome-wide significance threshold. Second part exhibits the 0.1-Mb region on each side of the peak SNP and its position is indicated by a vertical red line with red triangle. The bottom of each panel shows the annotated genes of the 200-kb region. Significant SNP that surpassed the threshold is indicated by red plots and peak SNP is indicated by purple plots. The dotted lines with arrows represent the traits related to the peak SNP. Gene is indicated by black boxes. If the significant SNP was located in the gene, the gene name is highlighted in red bold font and the coding region of the gene was indicated by black vertical lines.
